# Welfare effects of weather variability: Multi-country evidence from Africa south of the Sahara

**DOI:** 10.1371/journal.pone.0206415

**Published:** 2018-11-28

**Authors:** Beliyou Haile, Sara Signorelli, Carlo Azzarri, Timothy Johnson

**Affiliations:** 1 International Food Policy Research Institute, Washington D.C., United States of America; 2 Paris School of Economics, Paris, France; 3 Duo Security, Ann Arbor, Michigan, United States of America; Universidade da Coruna, SPAIN

## Abstract

Climate change and weather variability pose serious threats to food and nutrition security as well as ecosystems, especially when livelihoods depend heavily on natural resources. This study examines the effect of weather variability (shock) occurring up to three planting and growing season prior on per capita monthly household expenditure in rural Tanzania, Uganda, and Ghana. The analyses combine monthly temperature (1950–2013) and precipitation (1981–2013) data with data from several rounds of household surveys conducted between 1998 and 2013. Substantial spatial and temporal heterogeneity is documented in the incidence of shocks, with effects dependent on both the study and lag period considered. Analysis of short panel data shows the cumulative effect of above-average precipitation on expenditure to be negative in Uganda -while positive in Tanzania-, but the relationship does not persist when pooling survey data spanning over a decade. The evidence from pooled data suggests a positive association between above-average temperature (heat wave) and expenditure in (historically cooler) Uganda, with the opposite effect observed in (the relatively warmer) Tanzania. For Ghana, the association between heat wave and expenditure is positive. There is no evidence of heterogeneous effects along several dimensions, except by agro-ecological condition. Further research into the effects of shocks on more direct outcomes–such as agricultural practices, yields, and dietary intake–is therefore recommended to shed light on possible impact pathways and appropriate localized adaptation strategies.

## Introduction

Climate change and variability are having significant effects on biological and human systems. Income and expenditures of millions of agricultural households worldwide are permanently close to the poverty line, fluctuating above or below depending on the climate. These changes threaten food and nutrition security by impacting the availability, accessibility, and utilization of food as well as the stability of food supply. [1, 2] For example, drought and extreme heat waves between 1964 and 2007 reduced global cereal production by about 10%. [3] Climate-related factors are also strong predictors of crop and animal production, income, diseases and undernutrition in vulnerable regions of the world [4].

Increased frequencies, intensities, and duration of events such as heat stress, drought, and flooding are projected in the coming decades, with effects expected to exhibit significant spatial variation depending on altitude and irrigation coverage. [5, 6] These changes could have devastating effects in places like Africa south of the Sahara (SSA), a region that heavily relies on rain-fed agriculture and where market and institutional failures limit the set of coping and adaptation strategies. [7–9] In this regard, productive assets could play a crucial role in smoothing consumption either directly (e.g., slaughtering of cattle) or indirectly (e.g., through distress sales), as would access to safety nets and financial products. [10, 11]

Vulnerable households face not only increased risk of food and nutrition insecurity but may not be able to invest in productivity-enhancing technologies [12, 13] thereby perpetuating poverty. Recurring weather shocks force vulnerable households to adjust the portfolio of their livelihood strategies in favor of low-risk and low-return investments as well as diversify their income sources and labor allocation within and outside agriculture. [14, 15] These concerns have brought the issue of resilience and vulnerability to the forefront of the development agenda. Building resilience is among the core pillars of the Sustainable Development Goals, critical for eradicating extreme poverty and ensuring shared prosperity [16].

When measuring the effects of climatic changes and variability, the spatial and temporal variation relevant to the farming systems and livelihood strategies under consideration must be adequately captured. Important is also the adaptive capacity of systems that affects the extent of vulnerability. [4] In this regard, precipitation is a critical determinant of agricultural productivity and interannual yield variability, especially in the tropical and sub-tropical areas of SSA with limited irrigation coverage. [17] While irrigation has the potential to increase the region’s productivity by up to 50%, only about 6% of the total cultivated area is irrigated. [18] In such settings, limited precipitation negatively affects seed germination, plant growth, the quantity and quality of yields, and livestock, while excessive precipitation could lead to surface run-off and soil waterlogging, damages to crops, worsened phyto-pathological conditions, and overall logistical challenges [19].

Another critical determinant of plant development and productivity is surface temperature. Warmer conditions lengthen the growing season, but excessive heat causes high soil evapotranspiration, especially in already warm areas. [13] For example, each degree day with above 30 degree Celsius is found to reduce maize yield in Africa by 1%–1.7% depending on the drought condition. [1] Globally, a negative correlation has been documented between surface temperature and precipitation, with dry seasons having more sunshine and less evaporative cooling and wet seasons associated with cooler temperature [20].

In this study, we combine several rounds of household survey data with spatially-explicit monthly time series precipitation and temperature data to examine the immediate and longer-term effects of weather shocks. The study focuses on three countries in SSA—Uganda, Tanzania, and Ghana. These countries represent diverse agro-ecological conditions–from warm humid (Ghana) to a mix of warm sub-humid and warm arid/semi-arid tropics (Tanzania and Uganda). Focusing on 1960-2014, the average annual precipitation (in millimeters) for Uganda, Tanzania, and Ghana was 1228, 1023, and 1180, respectively, with a coefficient of variation of 15, 18, and 16, respectively. [21, 22] Uganda, Tanzania, and Ghana are ranked the 17^th^, 27^th^, and 93^rd^, respectively, in the world in terms of vulnerability to extreme weather events, including droughts, flooding, heat waves, and increased intensity of tropical cyclones. [23] While Ghana witnessed a relatively large decline in poverty over the last two decades, thanks to the structural shift it underwent, more than a third of Ugandans and Tanzanians lived below the poverty line of $1.90 a day in 2012/2013 (see supplemental material S1 Table for additional details about the study countries).

We estimate the effects of weather shocks on per capita monthly household consumption expenditure, our proxy for household welfare. Weather shocks could impact welfare through changes in farming systems (such as changes in cultivated area as well as crop and livestock mix), human and livestock health (such as pests and diseases), physical infrastructure (such as the destruction of roads and warehouses), and general equilibrium effects (such as reduced purchasing power due to weather-shock induced inflation). In addition, possible heterogeneity in the effects of shocks is also examined along several dimensions that could affect sensitivity and adaptive capacity–land holding, livestock wealth, durable assets, gender and education of the household head, as well as agroecology.

## Materials and methods

### Data and summary

Monthly time series precipitation data (1981–2013) are obtained from the Climate Hazards Group InfraRed Precipitation with Station data (CHIRPS). [24] Theses interpolated data have a 0.05° latitude by 0.05° longitude spatial resolution, a finer resolution than other long-term spatial precipitation datasets, and are based on both weather station and satellite data. Monthly time series temperature data (1950–2013) are obtained from the Center for Climatic Research (CRU) at the University of Delaware and have a 0.5° latitude by 0.5° longitude spatial resolution [25].

Repeated cross-sectional household survey data for Ghana come from three rounds of the Ghana Living Standards Survey (GLSS) (1998/99, 2005/06 and 2012/13). For Tanzania, we use two rounds of repeated cross-sectional data from the Tanzania Household Budget Survey (HBS) (2000/01 and 2006/07) and three rounds of panel data from the Tanzania National Panel Survey (NPS) (2008/09, 2010/11 and 2012/13). For Uganda, two rounds of repeated cross-sectional data from the Uganda National Household Survey (NHS) (2002/03 and 2005/06) and three rounds of panel data from the Uganda National Panel Survey (NPS) (2009/10, 2010/11 and 2011/12) are used. The analysis herein is restricted to rural agricultural households, who are expected to be impacted by weather variability the most and more directly. These datasets are used to construct a welfare measure–per capita monthly total (food and non-food) household expenditure in 2011 purchasing power parity (PPP). This outcome variable captures not only the direct effects of weather-induced yield variability, post-harvest losses, or changes in livestock and human health but also general equilibrium effects due to shock-induced price changes.

Guided by the empirical literature, we construct several socioeconomic and biophysical conditioning variables. These include household land holding (in hectares); livestock wealth (in Tropical Livestock Units–TLU); two indices of wealth constructed through factor analysis (iterated principal factor approach) based on ownership of durable assets and the quality of dwelling condition; the age, gender and education of household head; as well as the following proxies of agricultural potential and infrastructure development: night lights, population density, and agro-ecological zones (AEZ). Annual satellite data on night lights come from the National Oceanic and Atmospheric Administration (NOAA) and are measured at 30 arc-second grids. Intensity of night lights is considered a good proxy of economic activity and infrastructure development. [26] Population density is measured at 2.5 arc-minute grids [27] while district-level AEZ data are constructed following FAO/IIASA methodology [28] (see supplemental material S2 Table for descriptive summary).

The NPS data contain (modified) Global Positioning System (GPS) coordinates of sampled households, allowing us to match gridded data at the household level. About 0.8%—3% of the sample in the Uganda NPS, depending on the survey round, were too close to water bodies and did not properly overlay with the gridded weather data resulting in missing values. We replace missing values by the values for the nearest household. For all other surveys, gridded weather data are matched at the district level. To the extent there are significant intra-district heterogeneity in weather conditions, the latter approach could be prone to measurement errors.

Given the focus on rural farm households, we construct indicators of weather shock by focusing on the weather condition during maize planting and growing season (hereafter PG season) of the respective country. Considering the intra-country heterogeneity in agro-ecologies, the PG season is defined by region, following the crop calendar from the Food and Agriculture Organization of the United Nations. The PG months considered are as follows. For Ghana, June and July for Brong Ahafo, Northern, Upper East, and Upper West regions and March to July for all other regions. For Tanzania, December to May for Iringa, Lindi, Mbeya, Morogoro, Mtwara, Pwani, Rukwa, and Ruvuma regions and January to May for all other regions. For Uganda, April to July for Northern region and February to May for all other regions. Monthly precipitation and temperature data are processed using Python (version 3.6.2) and R (version 3.4.1), respectively, while Stata (version 15) and ArcGIS (version 10.3) software packages are used for the multivariate analyses and cartography, respectively.

**Fig 1:**
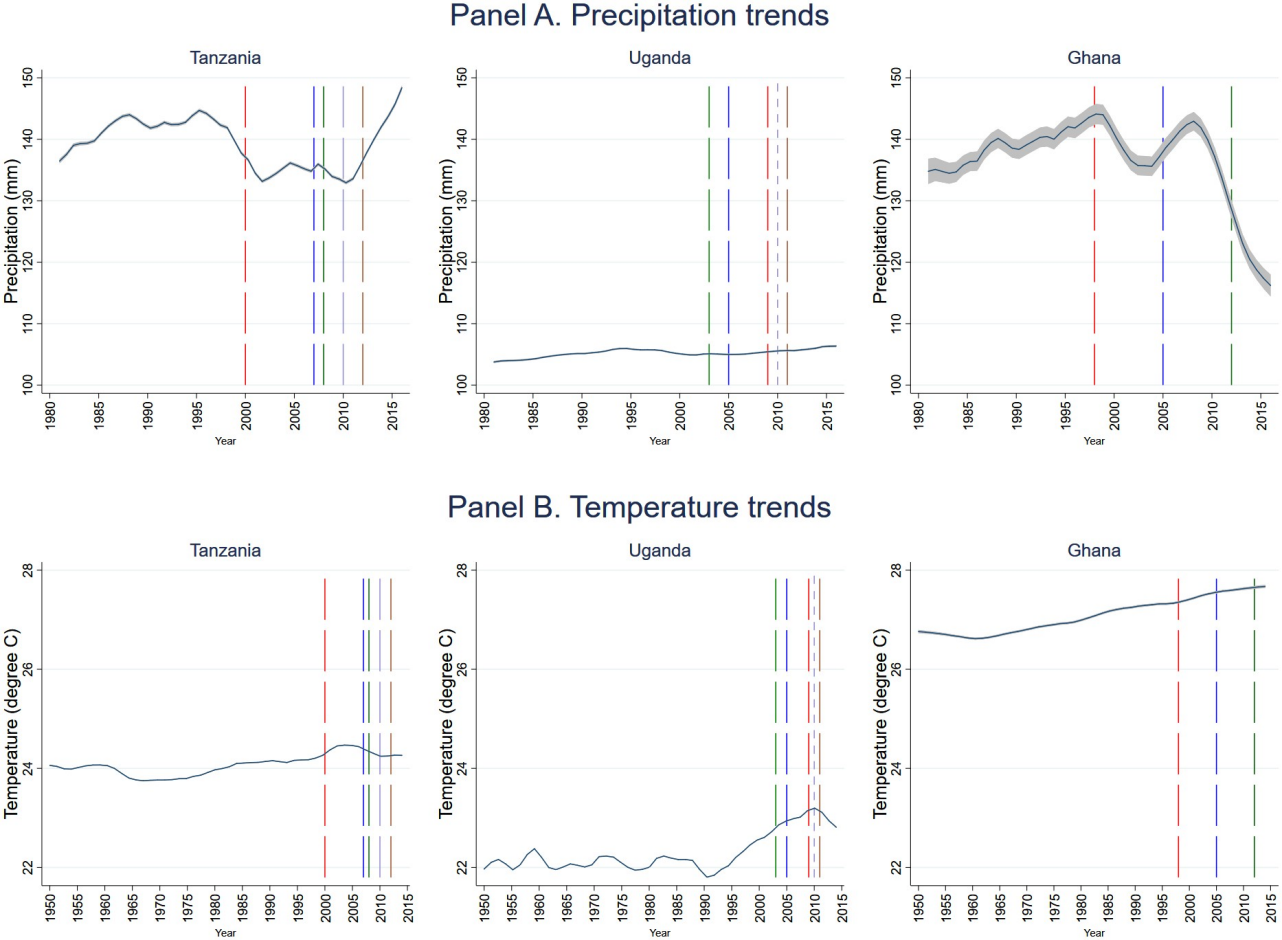
Trends in average monthly precipitation and temperature (by country). Panels A and B show trends in average monthly precipitation and temperature, respectively, for the planting and growing seasons of the respective country.

Fig 1 shows the average monthly precipitation and temperature for the PG seasons. Uganda shows a steadier rainfall regime, while Tanzania and Ghana experience cyclical patterns of lows and highs (Fig 1, panel A). A warming trend is observed across the board, with Uganda and Ghana scoring the coldest and warmest average temperature, respectively (Fig 1, panel B). Uganda’s temperature has increased by more than 1 degree Celsius (C) between 1990 and 2010 while Ghana’s temperature has risen at about 0.2 C per decade since 1960. Previous evidence shows Ghana to have experienced a shift towards a longer dry season, a vanishing wet season, and rising temperature [29, 30] while Tanzania and, in particular, Uganda have experienced both recurring dry spells and wet spells, depending on the region [31].

### Methods

We consider three lag periods –*L*1, *L*2, *L*3–to examine the immediate and longer-term effects of weather shocks. For households surveyed in year *t* and month *m*^*s*^ and where *m*^*s*^ falls *after* the beginning of the harvest season (*m*^*h*^), *L*1 measures the PG season in *t* − 1 while *L*2 and *L*3 measure the PG season in *t* − 2 and *t* − 3, respectively. If *m*^*s*^ falls *before* *m*^*h*^, *L*1 measures the PG season in *t* − 2 while *L*2 and *L*3 measure the PG season in *t* − 3 and *t* − 4, respectively. Defining lags like this is deemed necessary since the effects of weather shocks during the PG season in year *t* would not be captured by household (food) expenditure data collected in year *t* and month *m*^*s*^ if *m*^*s*^ preceded *m*^*h*^. For each *L*, variation in precipitation (*prec*) and temperature (*temp*) is measured by the number of months within the PG season when monthly *prec* (or *temp*) fell within different standard deviations (*SD*) above or below the long-term average (*LTA*) as shown in Eqs (1)–(4).

$$\begin{array}{c} W_{tm}^{LTA}=\frac{1}{NT}\sum_{t}\sum_{m}W_{tm}, \end{array}$$ (1)

$$\begin{array}{c} W_{tm}^{SD}=\sqrt{\frac{1}{NT}\sum_{t}\sum_{m}(W_{tm}-W_{tm}^{LTA}}{)}^{2}, \end{array}$$ (2)

$$\begin{array}{c} W_{tm}^{Posk}=\sum_{m}1_{W_{tm}>\left(W_{tm}^{LTA}+kxW_{tm}^{SD}\right)}, \end{array}$$ (3)

$$\begin{array}{c} W_{tm}^{Negk}=\sum_{m}1_{W_{tm}<\left(W_{tm}^{LTA}-kxW_{tm}^{SD}\right)}, \end{array}$$ (4)

where *W* is *prec* or *temp*; *t* and *m* (∀*m* ∈ *PG*) are year and month indices, with *T* (*N*) denoting the total number of PG seasons (months) from baseline (1950 for *temp* and 1981 for *prec*) to *t*. Superscripts *Pos* and *Neg* in Eqs (3) and (4) represent positive and negative shocks, respectively, with *k* (= 1, 2, 3) measuring standard deviation units by which *W*_*tm*_ is above or below $W_{tm}^{LTA}$. As discussed in [32], weather variability is measured in different ways including the change between two periods; the difference between *t*’s weather and *LTA*; based on indicators for whether *t*’s weather falls within different *SD* of *LTA*; and self-reported shock measures.

The way we define shocks allows us to better capture possible intra-PG weather variations that could affect plant growth and agricultural production, relative to shocks defined based on averages for the entire PG season. To examine the cumulative effects of shocks, we construct indicators based on the total number of months in *L*1, *L*2, and *L*3 that experience the respective shock. These indicators will also capture possible multi-year shocks that have occurred during the reference period. In terms of the welfare effects, if a household that faced shocks in, say, the preceding two seasons had already adapted its livelihood strategies, the marginal effect of the same shock occurring in the current season could be minimal, relative to its effects on households facing it for the first time *ceteris paribus*. On the other hand, if a recurring shock already depleted the coping capabilities of households, its marginal effect could be substantial. As such, the effect of cumulative shocks is an empirical matter.

Let moderate and extreme shocks represent a state where monthly temperature (or precipitation) falls, respectively, between 1-2 and above 2 *SD* of the *LTA* in absolute value. Let us also define below- and above-average precipitation shock by a dry and wet spell, respectively, and above- and below-average temperature shock by heat and cold wave, respectively. Focusing on just *L*1, and depending on the country and survey year, about 10% to 30% of the households experienced at least a month of moderate dry spell or wet spell each, with the highest incidence of the latter observed in Uganda (see supplemental material S1 Fig). The incidence of extreme dry or wet spell, especially above three *SD* of *LTA* (in absolute value) is not that common in our sample, especially in Tanzania. With regards to temperature, neither Uganda nor Ghana experienced cold waves during the reference period and less than 5% of Tanzanian households experienced at least one month of moderate cold waves. Heat waves were common, especially in Uganda, for all the years except 2012. The results for Uganda are consistent with the long-term wetting condition and the relatively sharp rise in temperature after 1990 summarized in Fig 1.

**Fig 2:**
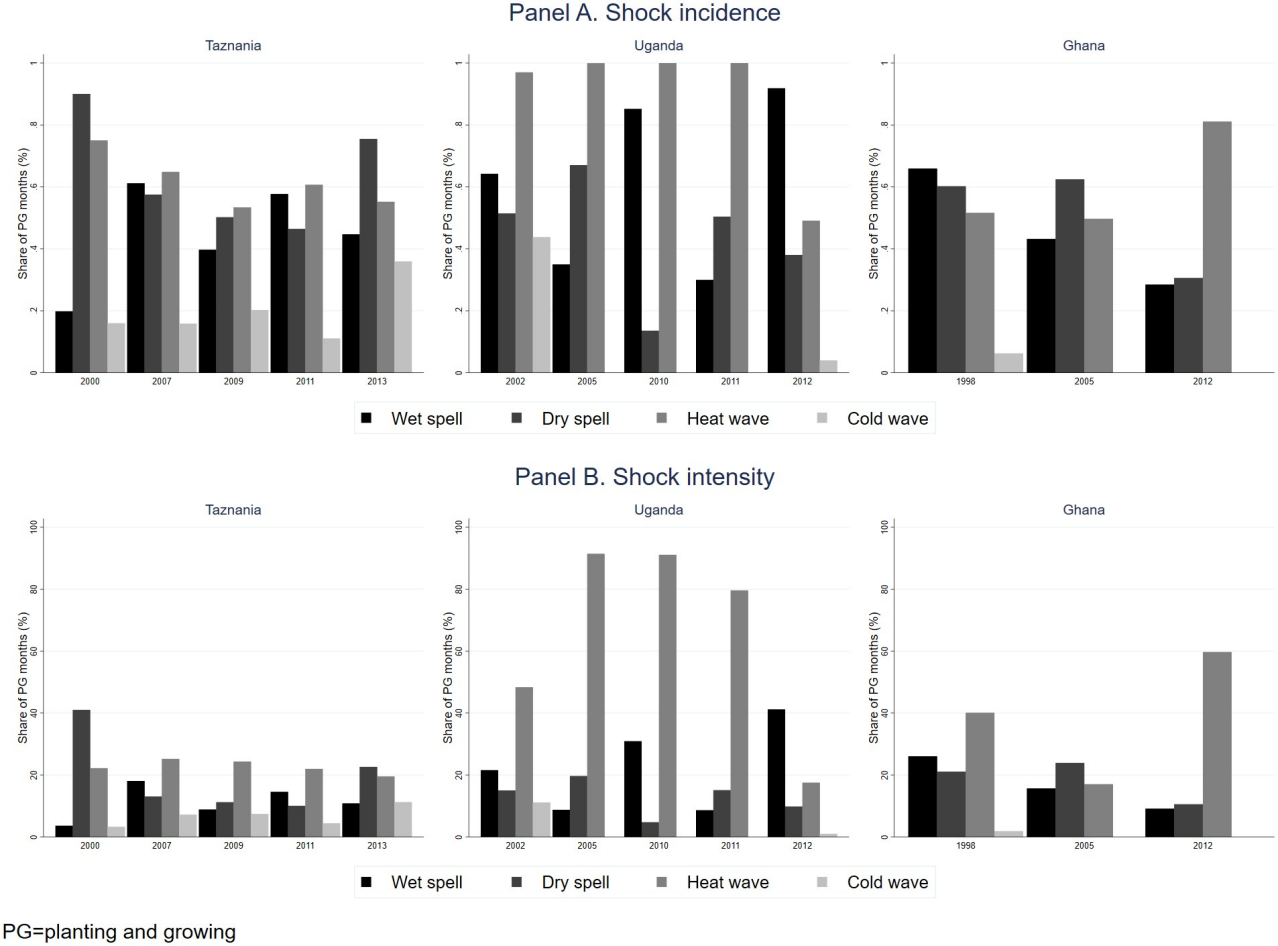
T-1 precipitation and temperature shocks (by country). Panel A shows the percentage of households experiencing at least a month of precipitation shock during the planting and growing season at T-1. Panel B summarizes the share of T-1 planting and growing season months exposed to the respective shock, conditional on survey households being exposed to one.

Considering the relatively small incidence of extreme shocks in our sample, we do not distinguish between moderate and extreme shocks in the subsequent analyses and define shocks based on above or below one *SD* of *LTA* cutoff. Fig 2 shows the percentage of households experiencing T-1 shocks (panel A) along with the intensity of shocks -conditional on experiencing one-measured by the share of PG months experiencing the respective shock (panel B) using pooled data. There is a significant temporal and cross-country variations in shocks, with Ghana showing a rise in heat waves and a decline in wet spells. Overall, Uganda has the highest incidence of wet spells and heat waves, suggesting the co-existence of the two shocks. These trends generally persist when examining longer lag periods (not shown). Due to the relatively low incidence of cold waves in our sample, we focus on dry spell, wet spell, and heat wave.

**Fig 3:**
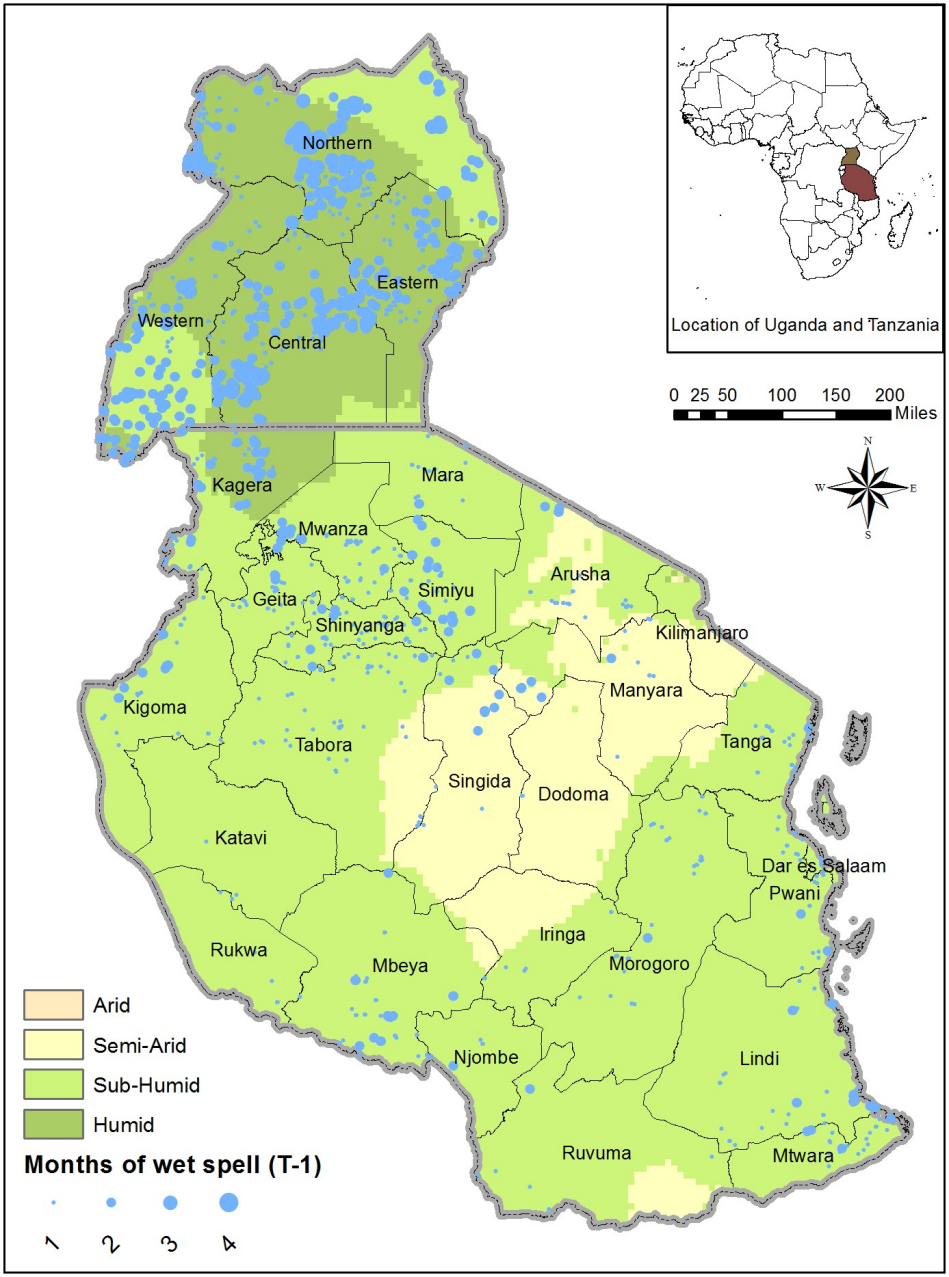
Spatial distribution of T-1 wet spell (Uganda and Tanzania). Reported are the number of T-1 planting and growing season months experiencing wet spell. The bigger the circle, the higher the number of months experiencing wet spell.

**Fig 4:**
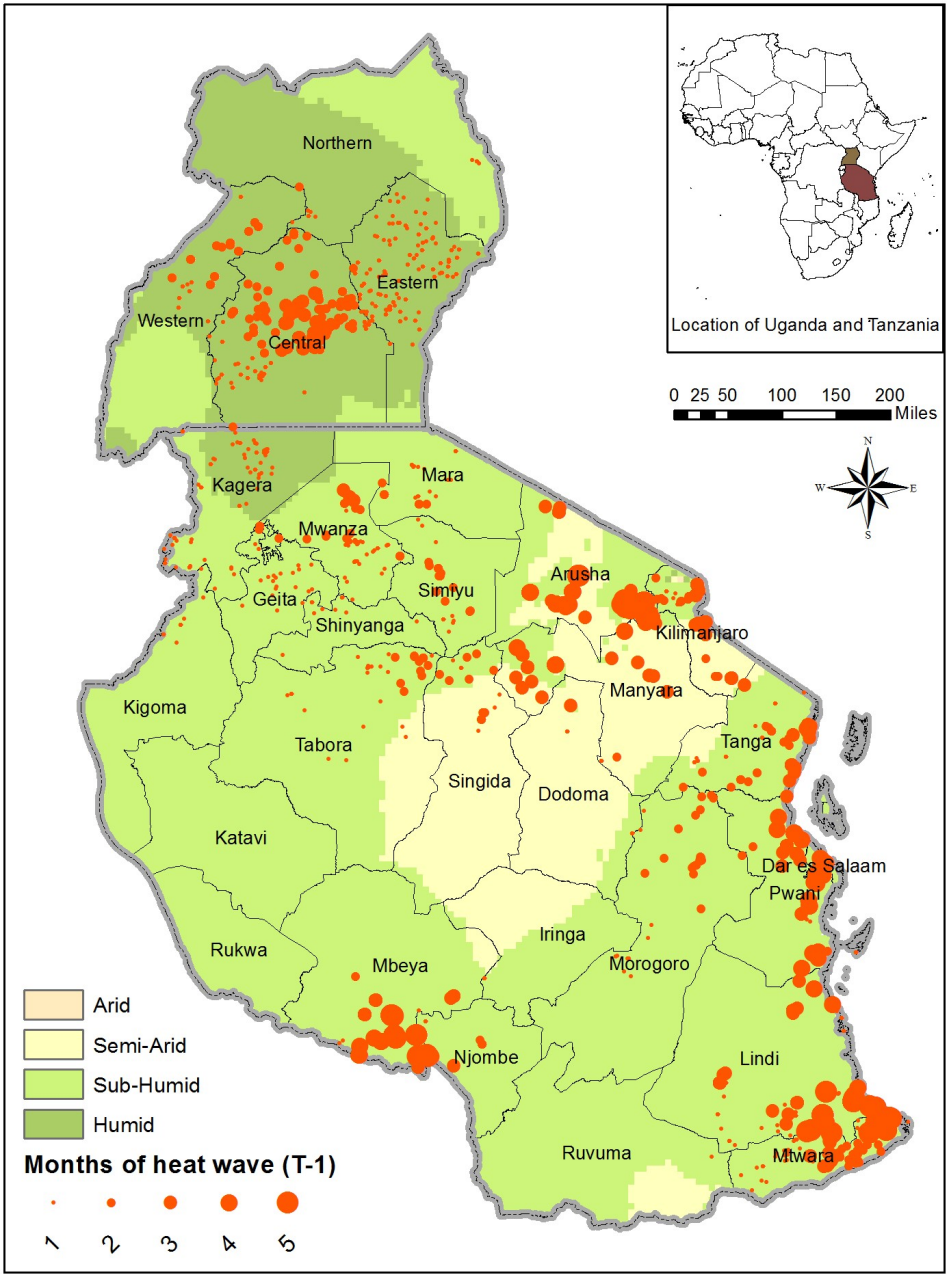
Spatial distribution of T-1 heat wave (Uganda and Tanzania). Reported are the number of T-1 planting and growing season months experiencing heat wave. The bigger the circle, the higher the number of months experiencing heat wave.

Figs 3 and 4 display the spatial distribution of T-1 wet spell and heat wave, respectively, for Tanzania and Uganda based on data from the third round of the NPS. Most of the humid areas of Uganda and parts of its subhumid Western region experienced wet spells while limited incidence of wet spells is observed in north and parts of the coastal areas of Tanzania. The humid parts of Uganda and coastal regions of Tanzania also experienced relatively high incidence of heat wave. The spatial distribution of *T* − 1 dry spell shown in supplemental material S2 Fig shows a higher incidence of dry spell in Tanzania, relative to Uganda.

**Fig 5:**
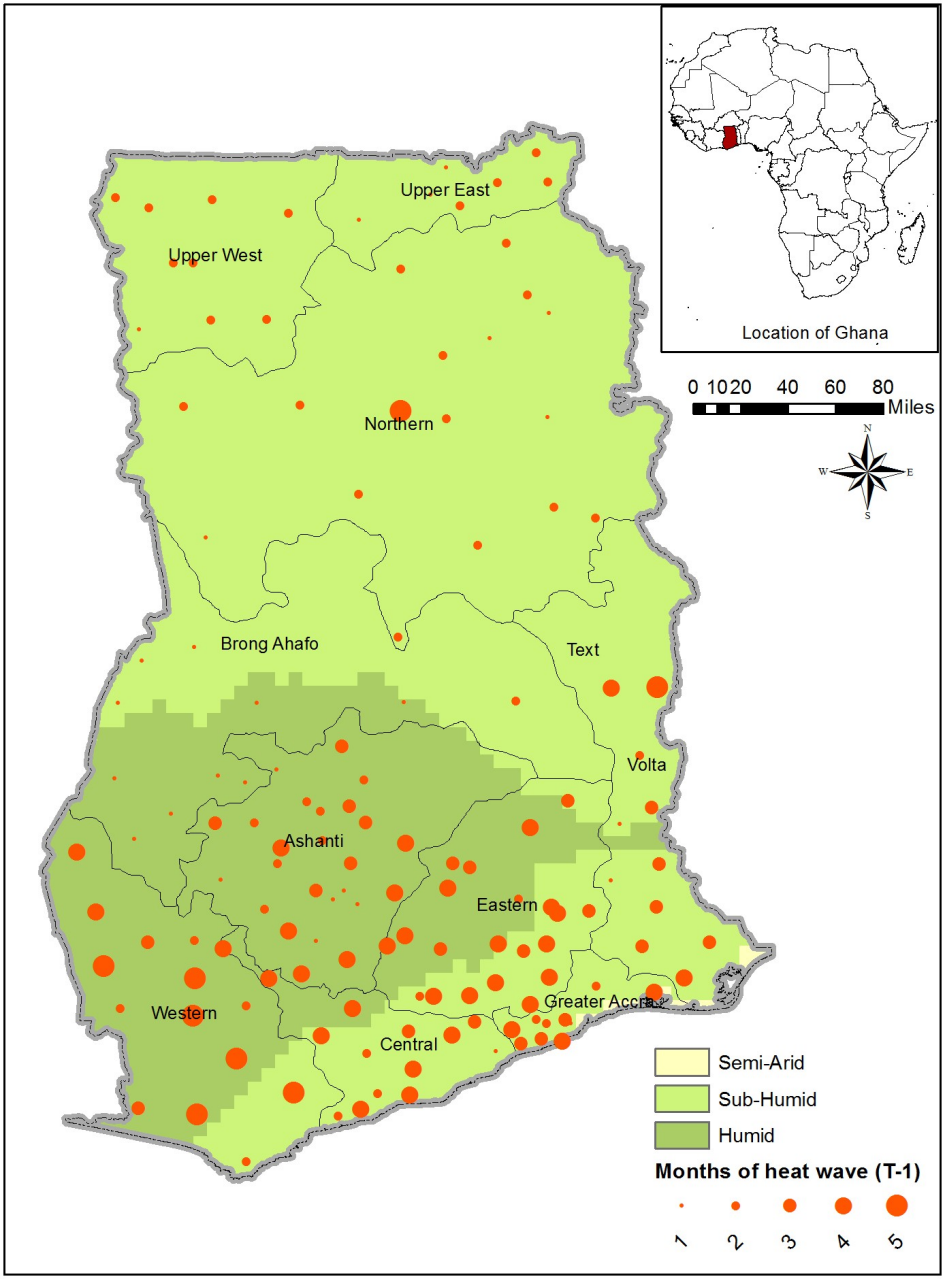
Spatial distribution of T-1 heat wave (Ghana). Reported are the number of T-1 planting and growing season months experiencing heat wave. The bigger the circle, the higher the number of months experiencing heat wave.

The spatial distribution of T-1 heat wave for Ghana based on data from round 6 of the GLSS shows a higher incidence and intensity of heat wave in the southern part (Fig 5). To recall, weather data for Ghana are aggregated at a district level potentially masking intra-district weather variability. Supplemental materials S3 and S4 Figs show the spatial distribution of *T* − 1 precipitation shocks for Ghana, which were relatively rear during the reference period.

These temporal and spatial summaries are consistent with previous findings, that reveal significant heterogeneity in rainfall patterns throughout Africa, with steadier rainfall and long-term wetting in East Africa and multi-decadal rainfall variability in other regions (especially the Sahel and South-East Africa). [33] As noted before, just 6% of the total cultivated area in SSA is irrigated, in sharp contract with the irrigation coverage of, for example, Asia and Latin America–37% and 14%, respectively. [18] As such, PG season drought could have detrimental effects on agricultural production and livelihoods. At the same time, wet spells can degrade soil nutrients and constrain productivity; damage vital infrastructure; and increase the incidence of toxins and pathogens. [34] The yield loss from wet spells might be especially high in areas already degraded due to unsustainable farming practices, vanishing natural vegetation cover, and urbanization [35].

For the multivariate analysis, we first use panel data from Tanzania and Uganda to estimate the individual-specific effects model in Eq (5), separately by country and lag period.

$$\begin{array}{c} \begin{array}{c} Y_{ht}=\alpha_{0}+\alpha_{1}prec_{ht}^{Pos1}+\alpha_{2}prec_{ht}^{Neg1}+\alpha_{3}prec_{ht}^{LTA}+\alpha_{4}temp_{ht}^{Pos1}+\\ \alpha_{5}temp_{ht}^{LTA}+\beta^{\prime }X_{ht}+\gamma^{\prime }Z+\eta_{h}+\epsilon_{ht} \end{array} \end{array}$$ (5)

where *h* and *t* are indices for household and period (*t* = 1, 2, 3); *Y* is (ln) per capita monthly expenditure (2011 PPP $); *prec*^*Neg*1^ (*prec*^*Pos*1^) measures the number of PG months with monthly precipitation under (over) one standard deviation below (above) long-term average value (*LTA*); *temp*^*Pos*1^ measures the number of PG months with monthly temperature over one standard deviation above *LTA*, *prec*^*LTA*^ and *temp*^*LTA*^ are long-term averages as defined before; the matrix **X** contains the socioeconomic variables summarized in supplemental material S2 Table. The matrix **Z** contains fixed effects for region and agro-ecological zones (AEZ), considering the spatial heterogeneity in shocks we find. Household-specific effects are captured by *η*; and model error term is given by *ϵ*, assumed to be independently and identically distributed (i.i.d).

When estimating the effects of *L*2 shocks, we in addition control for weather condition in *L*1. Similarly, the average weather condition for *L*1 and *L*2 is controlled when estimating the effects of *L*3 shocks. Estimates of *α*_*s*_(*s* = 1, 2, 4) measure the partial effects (in percentage points) of a month increase in the duration of the respective shock. When fitting the model with *L*1 shocks, the *α*_*s*_ ∀*s* mostly capture the immediate effect of shocks through, for example, yield reduction, crop failure, loss of livestock, or weather-induced morbidity. With longer lag periods, the *α*_*s*_ capture, in addition, any lingering effects of shocks on agricultural productivity in subsequent seasons due to, for example, limited use of improved technologies, reduced labor productivity, or inflationary pressure caused by production shortfall. If $|\alpha_{s}^{L3}\left|<\right|\alpha_{s}^{L2}\left|<\right|\alpha_{s}^{L1}|$ (∀*s*), the effect of shock dissipates over time.

If *Cov*(**X**_*ht*_, *η*_*h*_) = **0** (∀*t*), Eq (5) can be estimated consistently using random effects (hereafter RE). Otherwise, the less efficient but consistent fixed effects (hereafter FE) estimator should be used to correct for bias due to unobservable, time invariant, individual-level heterogeneity. [36, 37] On the other hand, FE point estimates could be highly inefficient and unreliable for conditioning variables with relatively low longitudinal variance. [38] Due to the relatively short panel data we analyze, the temporal variation of some of the conditioning variables is limited. For the sake of comparability, we estimate Eq (5) using both the FE and RE estimators, along with tests of overidentifying restrictions. [39] All panel regressions report cluster-robust standard errors -as observations are clustered at the household level- to correct for intra-household serial correlation and cross-sectional heteroscedasticity. [40] We progressively increase the elements of **X** and **Z** to examine the sensitivity of estimates, but only report the results from the most parsimonious specification for brevity. Results from the less parsimonious specifications, available upon request, are qualitatively similar.

Taking advantage of the rich dataset we have complied, we complement panel analysis with two more identification strategies. First, we analyze pooled data from all the available surveys–spanning up to 14 years–using weighted least squares (WLS hereafter) estimator, calculating robust standard errors clustered at the year-district level. In addition, we analyze pooled data using pseudo-panel method. Pseudo-panel analysis of repeated cross-sectional independent surveys with comparable methodology and reference population helps mitigate the limitations of short panel and pooled cross-sectional data. [41] The pseudo-panel approach can be formalized as shown in Eq (6).

$$\begin{array}{c} \begin{array}{c} {\bar{Y}}_{ct}=\alpha^{\prime }{\bar{W}}_{ct}+\beta^{\prime }{\bar{X}}_{ct}+\gamma^{\prime }{\bar{Z}}_{ct}+{\bar{\eta }}_{ct}+{\bar{\epsilon }}_{ct} \end{array} \end{array}$$ (6)

where *c* is the cohort index (*c* = 1, …, *C*) and *t* is time (*t* = 1, …, *T*), with the total number of observations given by *C*x*T*; the matrix **W** contains all the weather variables from Eq (5); the other matrices are as defined before; *η*_*ct*_ is the cohort fixed effect; and *ϵ*_*ct*_ is the model error term. For each $k\in \left(Y,W,X,Z\right),{\bar{k}}_{ct}=1/n_{ct}\sum_{h=1}^{n_{ct}}k_{ht}\left(\forall \;c,t\right)$, where *n*_*ct*_ is the number of households in cohort *c* at time *t*. While *η*_*ct*_ is time invariant in the population, given that population cohorts contain the same units across all *t*’s, it is unlikely to be time invariant in sample-based cohorts [42].

The manner in which synthetic cohorts are constructed bears an implication on standard errors and unobserved heterogeneity, since sample-based cohort averages represent an approximation of the true cohort population values. [42, 43] Large (small) *n*_*c*_ minimizes (increases) standard errors, but it increases (decreases) the efficiency loss, with the consequent need to balance the bias-efficiency tradeoff. Cohorts should be defined based on time-invariant variables and should allow enough temporal and cross-sectional variation in the true cohort mean for parameter identification. [44] As such, the determination of *n*_*c*_ (and hence *C*) is an empirical question to be determined based on the data generating process. In our case, and given the spatial aggregation in weather data, cohorts are defined using district of residence.

Alternative pseudo-panel estimators, with varying assumptions for deriving large sample approximations, are available including the efficient Wald (EWALD) [45], the error-in-variables estimator (EVE) [43], and the unbiased error-in-variables estimator (UEVE). [46] The EWALD estimates Eq (6) controlling for cohort fixed effects, although it might be biased and inconsistent when sample size is small and temporal variation in cohort characteristics is limited. [46] On the other hand, the EVE, shown to be consistent with large *t* [42], estimates the variances and covariances of the sampling errors from individual observations and subsequently applies the standard error-in-variables procedure.

The UEVE, shown to be approximately unbiased in small samples and to have smaller finite sample variance than the EVE [46], makes the sample variance adjustment factor dependent not only on the numbers of cohorts but also on the model parameters. In this study, the UEVE is used to estimate Eq (6). As can be seen from supplemental material S5 Fig, the median *n*_*c*_ ranges between 25 and 75 for Uganda and Tanzania, and 20 and 40 for Ghana, depending on the survey year. We therefore weigh cohort averages by ${\sqrt{n}}_{c}$ to mitigate heteroscedasticity. [43] We first apply the UEVE estimator to the true panel data, treating them as though they were repeated cross-section to test the performance of UEVE estimates using FE estimates as a benchmark. Next, the UEVE is applied on the pooled data.

Finally, we examine possible differential cumulative effects of dry spells and heat waves on household expenditure along the following policy relevant dimensions: gender and education of the household head, household wealth –measured by land size, livestock wealth, and ownership of durable assets–, and agro-ecology. Differential effects are estimated by including interaction terms of the relevant variables in Eq (5).

## Results and discussion

**Fig 6:**
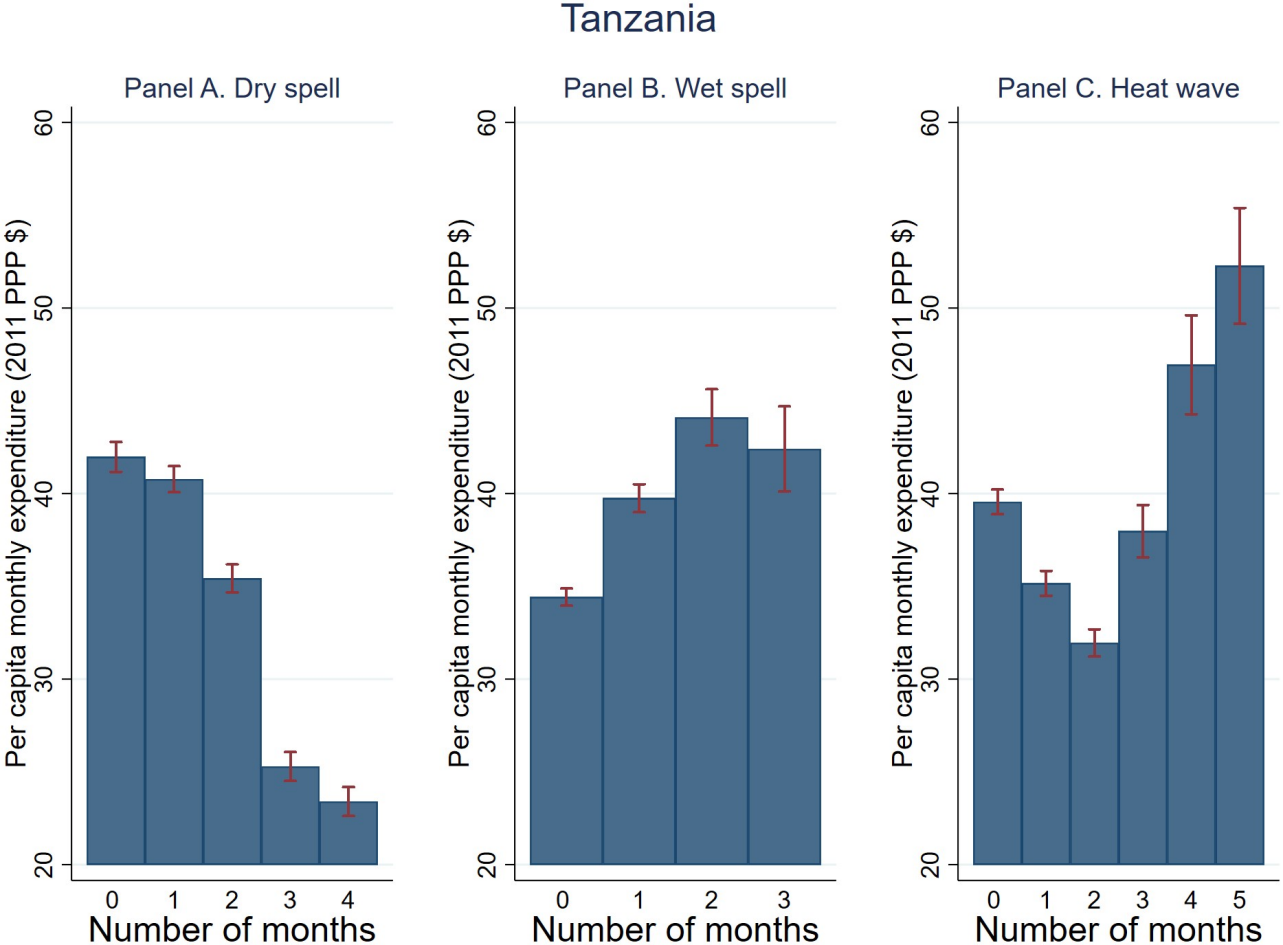
Weather shocks and per capita monthly expenditure (2011 PPP $) (Tanzania). Plotted are average per capita monthly expenditure and 95% confidence bands against T-1 planting and growing season months that experienced dry spell, wet spell, and heat wave.

**Fig 7:**
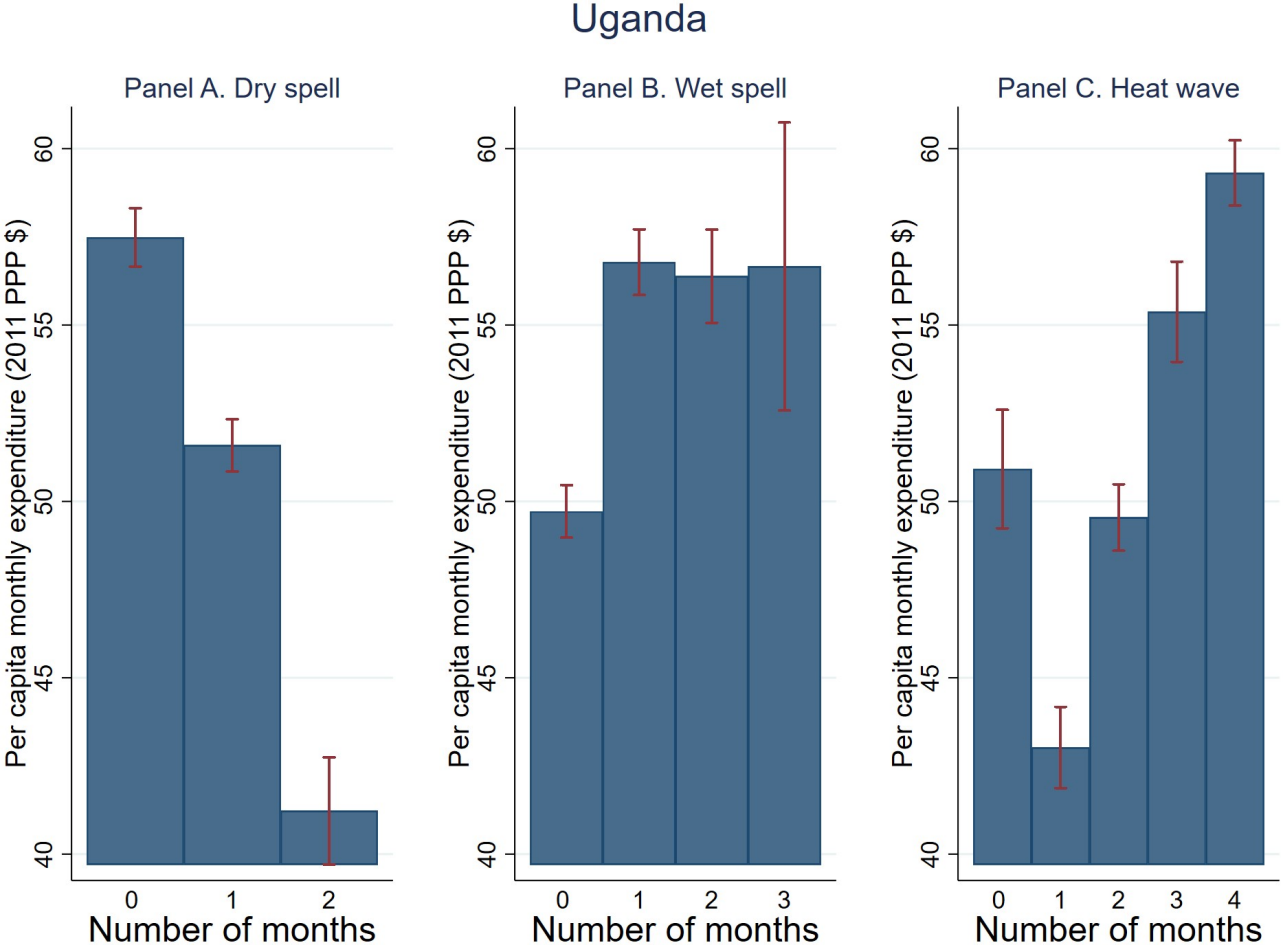
Weather shocks and per capita monthly expenditure (2011 PPP $) (Uganda). Plotted are average per capita monthly expenditure and 95% confidence bands against T-1 planting and growing season months that experienced dry spell, wet spell, and heat wave.

**Fig 8:**
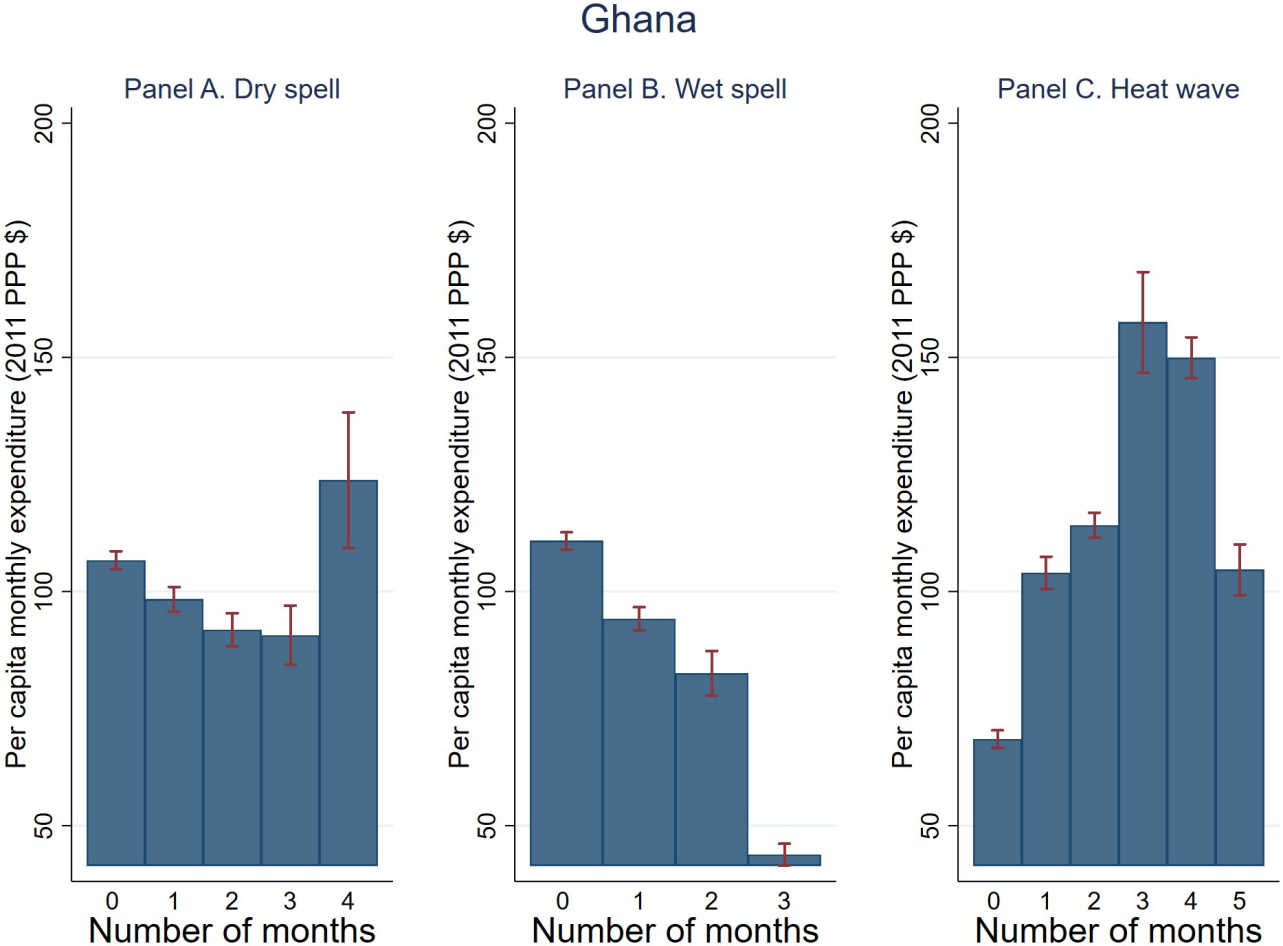
Weather shocks and per capita monthly expenditure (2011 PPP $) (Ghana). Plotted are average per capita monthly expenditure and 95% confidence bands against T-1 planting and growing season months that experienced dry spell, wet spell, and heat wave.

The unconditional relationship between household expenditure and the number of T-1 PG months experiencing shocks based on pooled data is shown in Figs 6–8. Dry spell is negatively correlated with expenditure across the board, with stronger association observed for Tanzania and Uganda (Figs 6 and 7). The association between wet spell and heat wave on the one hand and expenditure on the other appears to vary by country. Specifically, for Uganda and Tanzania, wet spell is positively associated with expenditure while the association between heat wave and expenditure appears U-shape. For Ghana on the other hand, both dry and wet spell are negatively associated with expenditure with an inverted-U relationship observed for heat wave. These trends generally persist when focusing on the cumulative (*T* − 1 to *T* − 3) shocks, with the exception of Tanzania where the association between wet spell and expenditure becomes negative (not shown).

RE estimates in supplemental material S3 Table show that Sargan-Hansen test statistics (S-H Chi) reject the null of orthogonal individual effects for both Uganda and Tanzania suggesting inconsistency in the RE estimates. Indeed, comparison of RE estimates with FE estimates in Table 1 reveals qualitative and quantitative differences. For example, while the FE estimates of the coefficients of long-term temperature are negative for both countries, their RE counterparts are either positive (for Uganda) or insignificant (for Tanzania). We therefore focus on FE estimates in the subsequent discussion. Historically wet areas of Uganda are associated with lower per capita monthly expenditure (Table 1, columns 1–4) possibly due to excess rain-induced flooding and loss of crops, as has previously been reported [47]. The correlation between long-term precipitation and expenditure is insignificant in Tanzania (Table 1, columns 5–8).

**Table 1:** Fixed effects estimates of the effects of shocks on (ln) per capita monthly expenditure (2011 PPP$).

	Uganda				Tanzania			
	T-1	T-2	T-3	Cum.	T-1	T-2	T-3	Cum.
	(1)	(2)	(3)	(4)	(5)	(6)	(7)	(8)
Dry spell	-0.042*** (0.016)	0.069*** (0.012)	-0.017 (0.011)	0.021** (0.010)	0.083*** (0.011)	0.014 (0.012)	-0.017 (0.012)	0.031*** (0.008)
Wet spell	0.043*** (0.011)	-0.028** (0.014)	-0.081*** (0.018)	-0.026** (0.012)	0.019 (0.013)	0.024* (0.013)	0.033** (0.013)	0.037*** (0.009)
LTA precipitation	-0.025* (0.013)	-0.026** (0.013)	-0.058*** (0.018)	-0.058*** (0.017)	0.000 (0.001)	0.002* (0.001)	0.001 (0.001)	-0.000 (0.001)
Heat wave	0.038*** (0.007)	-0.022** (0.009)	0.024*** (0.008)	-0.005 (0.008)	-0.005 (0.006)	-0.001 (0.008)	0.024*** (0.008)	0.004 (0.006)
LTA temperature	-0.361** (0.180)	-0.458** (0.202)	-0.568** (0.264)	-0.534** (0.237)	-0.025** (0.011)	-0.030*** (0.011)	-0.030*** (0.011)	-0.028** (0.011)
N	5,087	5,087	5,087	5,087	4,770	4,770	4,770	4,770
N_g	1,698	1,698	1,698	1,698	1,590	1,590	1,590	1,590
R2-within	0.217	0.228	0.218	0.210	0.107	0.094	0.097	0.100
SD of residuals	1.020	1.175	1.907	1.784	0.713	0.711	0.709	0.719
Fraction of FE var.	0.852	0.886	0.953	0.946	0.717	0.713	0.712	0.718
Corr. coef	-0.808	-0.856	-0.947	-0.939	-0.708	-0.707	-0.703	-0.715

Notes: Columns 1–4 and 5–8 show fixed effects estimates for Uganda and Tanzania, respectively, from the most parsimonious specification. Only the coefficients of the weather variables are reported. Columns 1 and 5 report effects of shocks occurring in the planting and growing (PG) season immediately before the survey year (T-1). Columns 2 and 6 report effects of shocks occurring in T-2 PG season. Columns 3 and 7 report effects of shocks occurring in T-3 PG season. Columns 4 and 8 report the cumulative effects of shocks, measured by the total number of months (T-1 to T-3) experiencing the respective shock. For each lag period, the conditioning variables Wet spell, Dry spell, and Heat wave refer to the number of PG season months experiencing the respective shock. LTA = long-term average, measured in millimeters (for temperature) and degree Celsius (for temperature). N = number of observations. N_g = number of household. SD = standard deviation. Robust standard errors clustered at the household level are in parentheses. Results are based on the balanced samples of rural agricultural households in the NPS data of the respective country.

*p<0.1,

** p<0.05,

*** p<0.01.

Historically warm regions of both countries are associated with lower expenditure, with a much stronger association observed in Uganda. Overall, FE results suggest that weather shocks matter more in Uganda than in Tanzania, with effects dependent on the lag period. Focusing on shocks during the planting and growing season immediately before the survey year (T-1), for example, a month increase in dry spell in Uganda reduces per capita monthly household expenditure by about 4% while one month increase in wet spell increases expenditure by about the same (Table 1, column 1). For temperature shocks, a month increase in T-1 heat wave is associated with a 3.7% increase in per capita expenditure in Uganda. When focusing on T-2 shocks on the other hand, each of these shocks have the opposite sign to what is found for T-1. Specifically, a month increase in T-2 dry spell in Uganda increases expenditure by about 7% while a month increase in T-2 wet spell or heat wave reduces expenditure by about 2%—3% (Table 1, column 2). When the reference period is T-3, wet spells (heat waves) appear to be negatively (positively) correlated with expenditure in Uganda (Table 1, column 3).

It is unclear what could potentially be driving this temporally non-linear effects of shocks. Existing evidence is generally mixed, with some [48] documenting no differential effects of climatic/weather variability while others [31, 49] document lag dependence. If households hit by a drought at *T* − 2 modify their production decision at *T* − 1, for example, by selecting high-yielding drought tolerant varieties and/or diversifying their income sources, a positive association between *T* − 2 dry spell and yields at *T* can be observed, controlling for *T* − 1 weather condition, *ceteris paribus*. Alternatively, lags in the adjustment of markets to weather variability could cause temporal dependence. A *T* − 2 drought that reduces *T* − 1 harvest may lead to a surge in post-harvest commodity prices possibly increasing household expenditure at *T*. The latter could especially be the case if there are not enough commodity stocks and imports to curtail inflationary pressure. Still, shocks may not affect expenditure significantly if there is consumption smoothing. Further research is needed to better understand possible general equilibrium implications of shocks and causal impact pathways. In terms of cumulative effects, we find dry (wet) spells to increase (reduce) per capita expenditure in Uganda by about 2% (Table 1, column 4), while both shocks have a positive effect on expenditure in Tanzania (Table 1, column 8).

WLS estimates summarized in Table 2 show a positive association between the cumulative number of dry spell and heat wave on the one hand and expenditure on the other for Uganda. For Tanzania, the cumulative effects of all the three shocks–dry spell, wet spell, and heat wave–on expenditure is negative. As noted before, and unlike the FE estimates, WLS estimates measure an association rather than causation due to possible omitted variables bias. Also, while FE results are based on interpolated gridded weather data matched with (modified) household GPS location, WLS results are based on shock indicators defined using a mix of household- (for panel waves) and district- (for non-panel waves) level weather data. As a result, the WLS estimates might be biased, especially in case of significant intra-district heterogeneity in weather condition, and should be interpreted with some caution.

**Table 2:** Weighted least squares estimates of the effects of shocks on (ln) per capita monthly expenditure (2011 PPP$).

	Uganda				Tanzania			
	T-1	T-2	T-3	Cum.	T-1	T-2	T-3	Cum.
	(1)	(2)	(3)	(4)	(5)	(6)	(7)	(8)
Dry spell	-0.123*** (0.025)	0.098*** (0.020)	0.073*** (0.019)	0.027** (0.012)	-0.115*** (0.016)	-0.081*** (0.017)	-0.059*** (0.017)	-0.077*** (0.011)
Wet spell	0.058*** (0.021)	-0.016 (0.019)	0.068*** (0.024)	0.009 (0.013)	0.031* (0.017)	-0.029* (0.017)	-0.139*** (0.016)	-0.055*** (0.012)
LTA precipitation	-0.000 (0.001)	-0.005*** (0.002)	0.002 (0.002)	-0.002 (0.001)	0.001** (0.001)	-0.004*** (0.001)	-0.003*** (0.001)	0.002*** (0.001)
Heat wave	0.084*** (0.011)	0.048*** (0.010)	0.055*** (0.012)	0.048*** (0.006)	0.004 (0.010)	-0.040*** (0.008)	-0.038*** (0.007)	-0.009* (0.005)
LTA temperature	-0.009 (0.011)	-0.007 (0.012)	-0.005 (0.013)	-0.024* (0.013)	0.018* (0.011)	0.016 (0.010)	0.011 (0.009)	0.014 (0.010)
N	17,398	17,398	17,398	17,398	17,434	17,434	17,434	17,434
R2	0.376	0.378	0.366	0.375	0.320	0.325	0.345	0.336

Notes: Columns 1–4 and 5–8 show weighted least squares estimates for Uganda and Tanzania, respectively, from the most parsimonious specification. Only the coefficients of the weather variables are reported. Please refer to the bottom of Table 1 for column notes and variable definition. Robust standard errors clustered at the year-district level are in parentheses. Results are based on pooled sample of rural agricultural households.

*p<0.1,

** p<0.05,

*** p<0.01.

Overall, the UEVE estimates in supplemental material S4 Table based on short panel data are different–both qualitatively and quantitatively–from FE estimates in Table 1. The former are either insignificant or, when significant, are associated to an opposite sign to the corresponding FE estimates. This finding might be due to the use of short panel data, which limits the extent of temporal variation in cohort averages, in turn causing aggregation bias. As summarized in Table 3, UEVE estimates based on pooled data spanning 14 years also differ from both FE and WLS estimates, especially for Uganda. Further research is needed to assess the appropriateness of a pseudo-panel approach to examine welfare effects of weather variability and to identify relevant dimension(s) for cohort construction that better fulfill the properties discussed earlier. While some consider a cohort size of 100–200 observations per cohort large enough, others note a cohort size of thousands of observations is needed to minimize bias. [44, 50] In our case, and as summarized in supplemental material S5 Fig, the median district-based cohort size ranges from 20 to 75 households, depending on country and survey round.

**Table 3:** Unbiased error-in-variables estimates of the effects of shocks on (ln) per capita monthly expenditure (2011 PPP$).

	Uganda				Tanzania			
	T-1	T-2	T-3	Cum.	T-1	T-2	T-3	Cum.
	(1)	(2)	(3)	(4)	(5)	(6)	(7)	(8)
Dry spell	-0.091** (0.036)	0.061 (0.041)	0.019 (0.036)	-0.007 (0.021)	-0.060* (0.032)	0.042 (0.159)	-0.029 (0.042)	-0.057*** (0.021)
Wet spell	0.050 (0.057)	-0.027 (0.026)	0.106** (0.047)	0.007 (0.026)	0.131 (0.085)	0.112 (0.234)	-0.091* (0.051)	-0.045 (0.066)
LTA precipitation	-0.002 (0.002)	-0.006** (0.003)	0.000 (0.004)	-0.003* (0.002)	0.001 (0.001)	-0.004 (0.003)	-0.009*** (0.003)	0.001 (0.001)
Heat wave	0.035 (0.065)	0.006 (0.021)	0.064* (0.034)	0.009 (0.017)	0.113* (0.063)	0.010 (0.123)	-0.140*** (0.028)	-0.023 (0.023)
LTA temperature	-0.034** (0.015)	-0.033** (0.016)	-0.046** (0.018)	-0.041** (0.017)	0.005 (0.019)	0.033 (0.022)	0.000 (0.016)	0.015 (0.019)
N	17,397	17,397	17,397	17,397	17,433	17,433	17,433	17,433
N_g	108	108	108	108	116	116	116	116
R2	0.521	0.568	0.562	0.534	0.600	0.507	0.690	0.612

Notes: Columns 1–4 and 5–8 show unbiased error-in-variables estimates for Uganda and Tanzania, respectively, from the most parsimonious specification. Only the coefficients of the weather variables are reported. Please refer to the bottom of Table 1 for column notes and variable definition. Robust standard errors clustered at the year-district level are in parentheses. Results are based on pooled sample of rural agricultural households.

*p<0.1,

** p<0.05,

*** p<0.01.

For Ghana, a consistent (across estimators and lag periods) result is found on the effects of above-average temperature (heat wave). Focusing on the cumulative effects (Table 4, columns 4 and 8), a month increase in heat wave is associated with about 14%-16% increase in per capita monthly household expenditure, depending on the estimator. Temperature variability in Ghana has previously been found to have a stronger effect on crop yields than rainfall variability. [51] As shown in Fig 1, Ghana has not only the warmest planting and growing season among the three countries, but has also experienced a consistent warming trend during the study period. The positive association between heat wave and expenditure could be due to confounding factors. As shown in Fig 5, heat wave occurred mostly in southern Ghana, a region with relatively low poverty rate. [35] WLS estimates also suggest a negative association between expenditure and the number of months of wet spells at *T* − 1. A negative correlation between annual rainfall and production of main staple crops has previously been documented, with above-average precipitation linked to an overflow of Ghana’s major water bodies and flooding, especially of communities close to the Volta River and in the relatively more degraded northern Ghana [52].

**Table 4:** Weighted least squares (WLS) and unbiased error-in-variables (UEVE) estimates of the effects of shocks on (ln) per capita monthly expenditure (2011 PPP$)–Ghana.

	WLS				UEVE			
	T-1	T-2	T-3	Cum.	T-1	T-2	T-3	Cum.
	(1)	(2)	(3)	(4)	(5)	(6)	(7)	(8)
Dry spell	-0.047 (0.045)	0.084** (0.041)	-0.168*** (0.062)	-0.020 (0.017)	-0.230*** (0.075)	0.053 (0.076)	-0.009 (0.129)	0.027 (0.038)
Wet spell	-0.226*** (0.046)	0.094** (0.045)	0.058 (0.036)	-0.043** (0.021)	-0.442*** (0.076)	0.131* (0.069)	0.066 (0.063)	0.033 (0.033)
LTA precipitation	0.004** (0.002)	0.005** (0.002)	-0.001 (0.003)	0.002 (0.002)	-0.000 (0.003)	0.010*** (0.003)	0.004 (0.004)	0.002 (0.003)
Heat wave	0.229*** (0.028)	0.279*** (0.021)	0.152*** (0.022)	0.137*** (0.008)	0.104*** (0.035)	0.330*** (0.035)	0.110** (0.050)	0.163*** (0.018)
LTA temperature	0.197** (0.090)	0.096 (0.077)	0.190** (0.095)	0.087 (0.066)	0.195** (0.090)	0.130 (0.084)	0.184* (0.098)	0.131 (0.079)
N	17,509	17,509	17,509	17,509	17,509	17,509	17,509	17,509
N_g					163	163	163	163
R2	0.469	0.521	0.440	0.541	0.550	0.647	0.465	0.653

Notes: Columns 1–4 and 5–8 show weighted least squares and unbiased error-in-variables estimates of the effects of shocks for Ghana, respectively, from the most parsimonious specification. Only the coefficients of the weather variables are reported. Please refer to the bottom of Table 1 for column notes and variable definition. Robust standard errors clustered at the year-district level are in parentheses. Results are based on pooled sample of rural agricultural households.

*p<0.1,

** p<0.05,

*** p<0.01.

The level of exposure to shocks as well as the speed and extent of recovery depend on livelihood strategies and assets, as well as on formal and informal institutions. Productive assets could help smooth consumption either directly (e.g., through slaughtering of livestock) or indirectly (e.g., through distressed sale), especially when access to financial products is limited. While relatively better-off farm households may be hit by weather variability the hardest, they may be more likely to recover the fastest considering their ability to mobilize and manage resources. [53] Also, some disadvantaged groups, with more severe constraints to productive resources and copying strategies, may be hit by shocks the hardest. [54] The full set of WLS parameter estimates reported in supplemental material S5 Table show several household-level variables to be significant. Across the board, household head education, land size, livestock wealth, durable assets, and the quality of dwelling condition all have positive effects on expenditure.

We find limited evidence of heterogenous effects of shocks along several dimensions considered. The expenditure-increasing effect of dry spells and heat waves appears to be marginally weaker in Uganda the higher the livestock assets and the larger the land size (see supplemental material S6 Fig). In addition, and relative to Ugandan households in humid agro-ecology, both drought and excess temperature seem to be negatively correlated with expenditure for those in subhumid agro-ecology. No differential effects of shocks is found for Tanzania (see supplemental material S7 Fig). For Ghana, drought in semi-arid areas reduces expenditure, relative to an insignificant association in humid areas, while a stronger positive association between expenditure and heat wave is observed in semi-arid areas, relative to humid areas (see supplemental material S8 Fig). These differential effects by agro-ecology may be explained by the generally inconsistent rainfall patters in dry agro-ecologies with relatively short length of growing period and, in many cases, with relatively consistent high temperature where the agro ecosystems are better adapted to harsh weather conditions.

Before concluding the paper, we acknowledge some limitations of the study. Gridded weather data are based on interpolation of weather station data (temperature) and a combination of weather station and satellite data (precipitation). As such, the accuracy of spatially interpolated climate variables is affected by several factors, including the density of weather station networks. District-level merging of gridded weather data for all surveys except the National Panel Surveys would also not capture intra-district weather variations resulting in possible measurement error. Related is the use of monthly (versus daily) weather data that masks potential intra-month and intra-day weather variations and their effects on agriculture and welfare. For example, the negative effect of a rise in temperature on maize yield is found to be stronger if it occurs during the day than at night. [1] When defining shocks, we relied on the maize planting and growing season, given that maize is the main staple crop in the region. Nonetheless, the overall effects of weather variability on the welfare of farm households is affected by the number of crops grown (and livestock owned) as well as the maturity time needed for each crop, in turn dependent on crop variety and genotypic traits. We are unable to disentangle the effects of these important determinants of both the sensitivity and adaptive capacity of farming systems and households.

## Conclusion

Climatic and weather variability pose a major challenge to the livelihoods of millions of farm households that heavily rely on rainfed agriculture. This study examines the short- and longer-term effects of weather variability based on nationally representative household survey data from rural Tanzania, Uganda, and Ghana. The three countries represent diverse farming systems and agro-ecological conditions with varying degree of vulnerability to extreme weather events. The effects of precipitation and temperature shocks during the maize planting and growing seasons are examined on per capita monthly household expenditure. Shock indicators are defined based on the number of months within the reference season where monthly precipitation (or temperature) was more (less) than one standard deviation unit above (below) the historical average, the latter defined based on time series data since 1950 (for temperature) and 1981 (for precipitation).

Consistently with previous literature, all countries, and Ghana in particular, experienced a warming trend over the last several decades with significant spatial (inter- and intra-country) and temporal heterogeneity in weather variability. Overall, the incidence of above-average precipitation (wet spell) has risen in Uganda but declined in Tanzania and Ghana. Relative to the other countries, a higher share of Ugandan households was exposed to above-average temperature (heat wave); the incidence of heat wave has risen in Ghana; and below-average temperature (cold wave) was more common in Tanzania. The effects of weather shocks on household expenditure depend on the country as well as the study and lag period considered, the latter non-linearly. In fact, we find a non-linear dependence of the effects of shocks on the lag period, suggesting possible general equilibrium effects.

Analysis of the cumulative effects of shocks based on pooled data covering over a decade shows both dry spells and heat waves to be positively correlated with household expenditure in Uganda, a country characterized by relatively cooler and humid agro-ecology, experiencing long-term wetting. Indeed, analysis of differential effects of shocks shows weaker expenditure increasing effects of these two shocks for Ugandan households living in sub-humid areas that already have shorter length of growing period. For Tanzania, on the other hand, both dry and wet spells, and to some extent heat waves, reduce household expenditure, with dry spells associated to the strongest negative effect. Ghana has experienced a shift towards a longer dry season, vanishing wet season, and a steady rise in temperature and we find a strong, persistent, and positive association between heat wave and expenditure, with suggestive evidence on the expenditure-reducing effect of wet spells.

We do not find strong evidence of heterogenous effects of shocks along several socioeconomic variables we examined except agro-ecology. Nonetheless, given the expected heterogeneity in the effects of climate change on yields based on latitude, irrigation coverage, and other agro-ecological conditions, a more targeted–spatially and temporally–analyses of the effects of weather shocks is recommended, preferably using finer resolution weather data. In addition, research on the links between the timing of shocks and household welfare considering both direct–through agricultural productivity and production–and indirect–through general equilibrium effects– is crucial to identify possible short- and long-term adaption options and causal impact pathways.

## Supporting information

S1 Table Profile of study countries. Cereal yield includes wheat, rice, maize, barley, oats, rye, millet, sorghum, buckwheat, and mixed grains. Production data on cereals relate to crops harvested for dry grain only.
(XLSX)

S2 Table Descriptive summary. Reported are weighted means and standard deviations (in parentheses). Column 1 shows summaries for Ghana. Columns 2 and 3 summarize data from Tanzania Household Budget Surveys (HBS) and National Panel Surveys (NPS), respectively. Columns 4 and 5 summarize data from Uganda National Household Surveys (NHS) and National Panel Surveys (NPS), respectively.
(XLSX)

S3 Table Random effects estimates of the effects of shocks on (ln) per capita monthly expenditure (2011 PPP$). Please refer the bottom of Table 1 for table notes.
(XLSX)

S4 Table Unbiased error-in-variables estimates of the effects of shocks on (ln) per capita monthly expenditure (2011 PPP$). For notes, please refer the bottom of Table 1. Results are based on panel sample of agricultural households from the NPS surveys of the respective countries.
(XLSX)

S5 Table Weighted least squares estimates of the effects of T-1 shocks on (ln) per capita monthly expenditure (2011 PPP$). For notes, please refer the bottom of Table 1. Results are based on data pooled across all the available surveys discussed in Section 1.
(XLSX)

S1 Fig Incidence of weather shocks by country, year, and intensity. Share of months of the planting and growing (PG) season immediately before the survey month exposed to varying intensity of weather shocks are reported. Legend shows monthly weather values falling within different standard deviation units of long-term average values.
(TIF)

S2 Fig Spatial distribution of T-1 dry spell in Uganda and Tanzania. Summarized are the number of planting and growing season months at T-1 experiencing dry spell. The bigger the circle, the higher the number of months experiencing dry spell.
(TIF)

S3 Fig Spatial distribution of T-1 dry spell in Ghana. Summarized are the number of planting and growing season months at T-1 experiencing dry spell. The bigger the circle, the higher the number of months experiencing dry spell.
(TIF)

S4 Fig Spatial distribution of T-1 wet spell in Ghana. Summarized are the number of planting and growing season months at T-1 experiencing wet spell. The bigger the circle, the higher the number of months experiencing wet spell.
(TIF)

S5 Fig Distribution of district-based cohort sizes (by country and survey year). N = number of districts.
(TIF)

S6 Fig Heterogenous effects of cumulative weather shocks on (ln) per capita monthly expenditure (2011 PPP $) (Uganda). Plotted are weighted least squares coefficient estimates of along with 95% confidence bands from the fully parsimonious model. Only the coefficients of the variables of interest are shown. Land is measured in hectares; “Headedu” refers to education level of the household head; livestock asset is measured in “TLU”; “Wealthy” refers to households in the top tercile of durable-assets based wealth index. “X” represents an interaction term and a statistically significant coefficient of an interaction term implies differential effect. The omitted category is humid agro-ecology.
(TIF)

S7 Fig Heterogenous effects of cumulative weather shocks on (ln) per capita monthly expenditure (2011 PPP $) (Tanzania). For notes, please refer to S6 Fig. The omitted category is sub-humid agro-ecology.
(TIF)

S8 Fig Heterogenous effects of cumulative weather shocks on (ln) per capita monthly expenditure (2011 PPP $) (Ghana). For notes, please refer to S6 Fig. The omitted category is humid agro-ecology.
(TIF)
