# Supplementary figures and images for: Welfare effects of weather variability: Multi-country evidence from Africa south of the Sahara
Source: PLoS One. 2018 Nov 28;13(11):e0206415. doi: 10.1371/journal.pone.0206415 (PMC6261409; doi:10.1371/journal.pone.0206415)

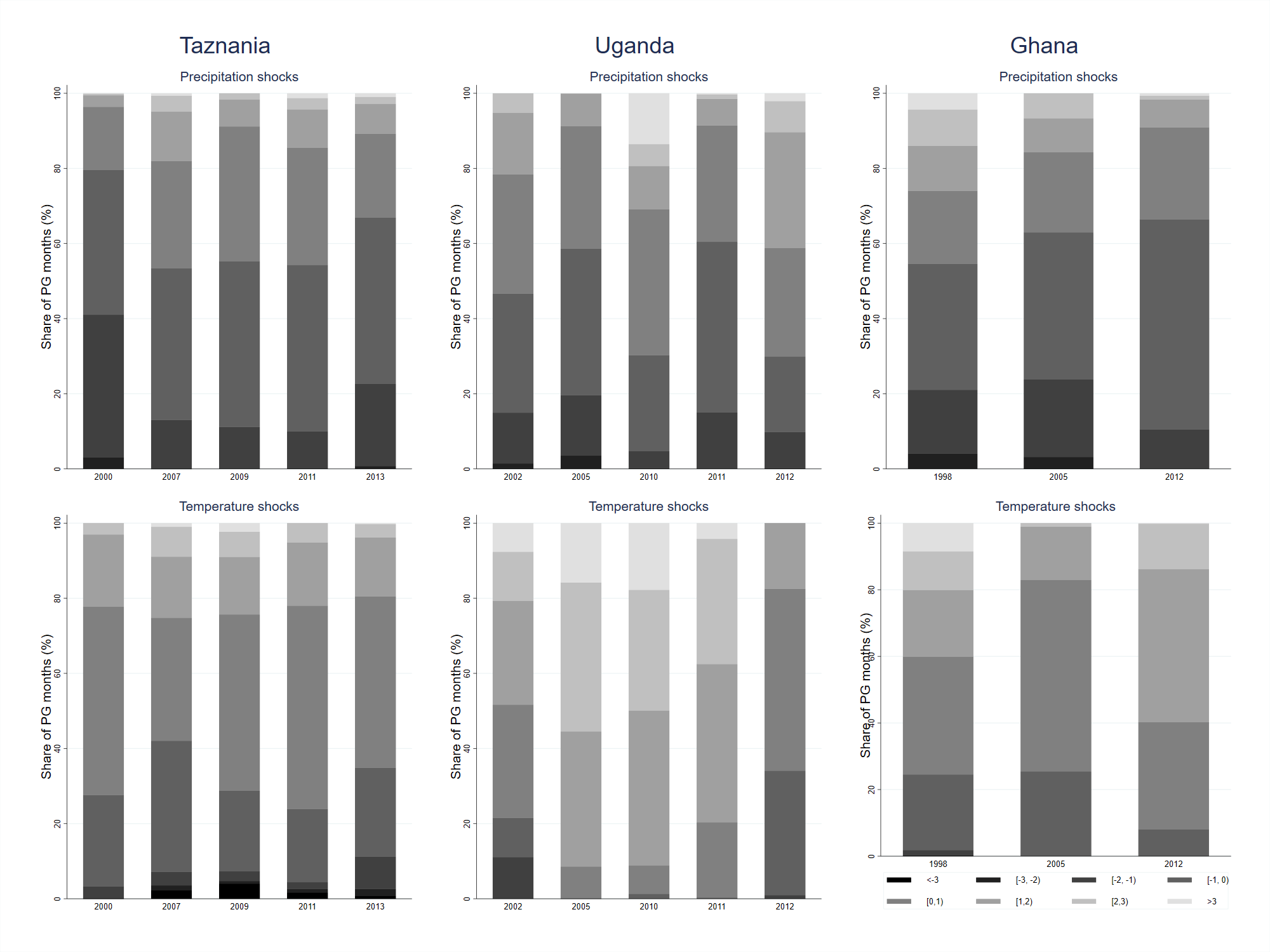

Supplement: S1 Fig — Share of months of the planting and growing (PG) season immediately before the survey month exposed to varying intensity of weather shocks are reported. Legend shows monthly weather values falling within different standard deviation units of long-term average values. (TIF) [file pone.0206415.s006.tif]

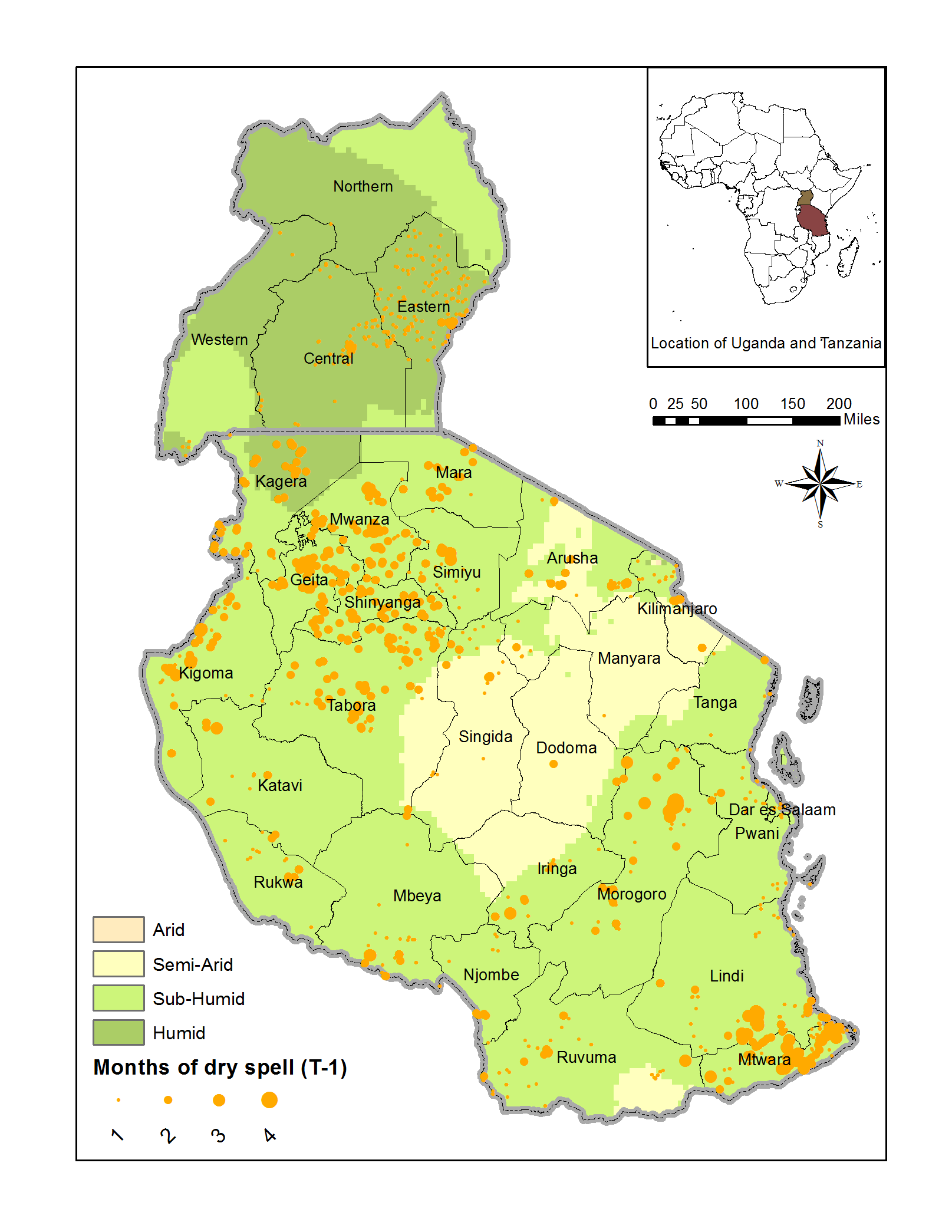

Supplement: S2 Fig — Summarized are the number of planting and growing season months at T-1 experiencing dry spell. The bigger the circle, the higher the number of months experiencing dry spell. (TIF) [file pone.0206415.s007.tif]

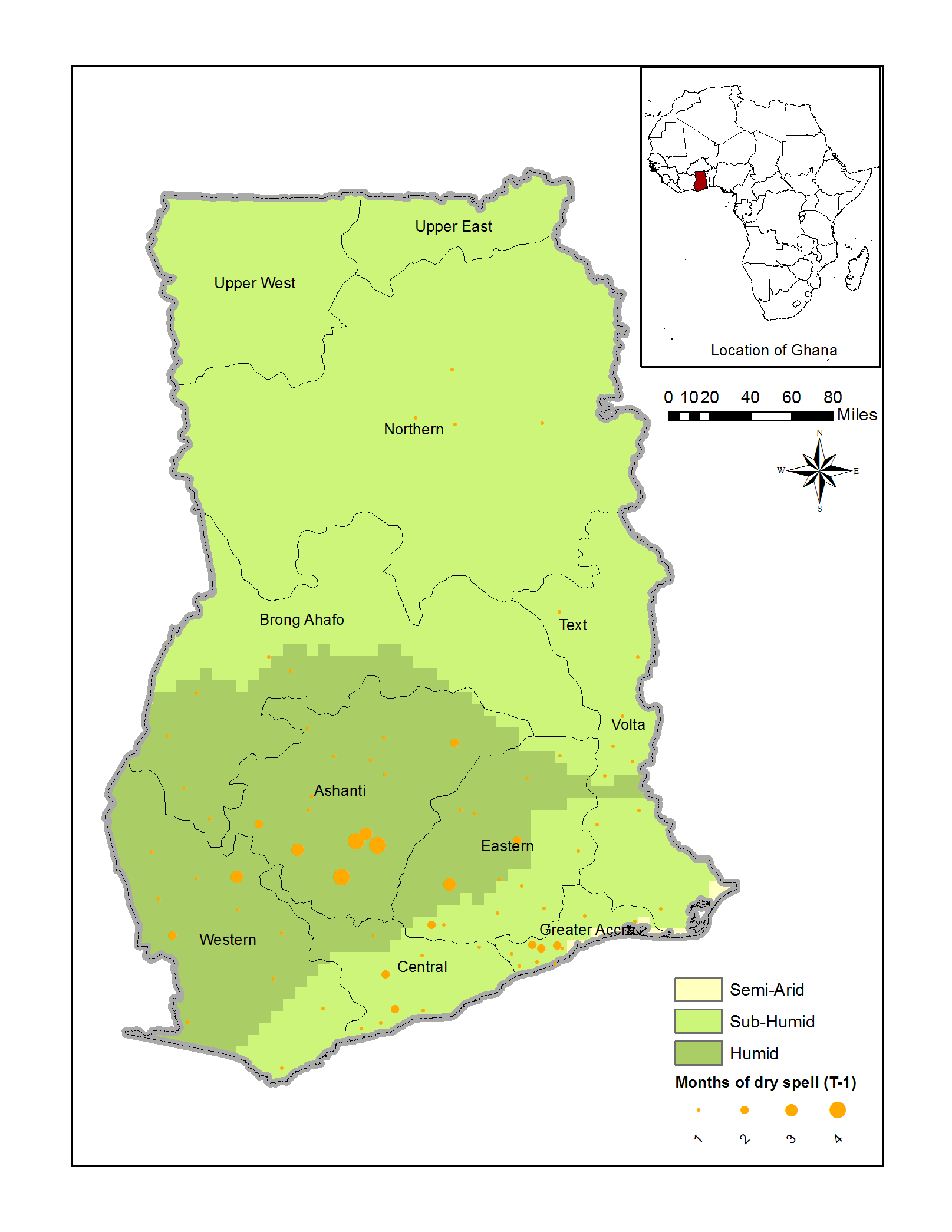

Supplement: S3 Fig — Summarized are the number of planting and growing season months at T-1 experiencing dry spell. The bigger the circle, the higher the number of months experiencing dry spell. (TIF) [file pone.0206415.s008.tif]

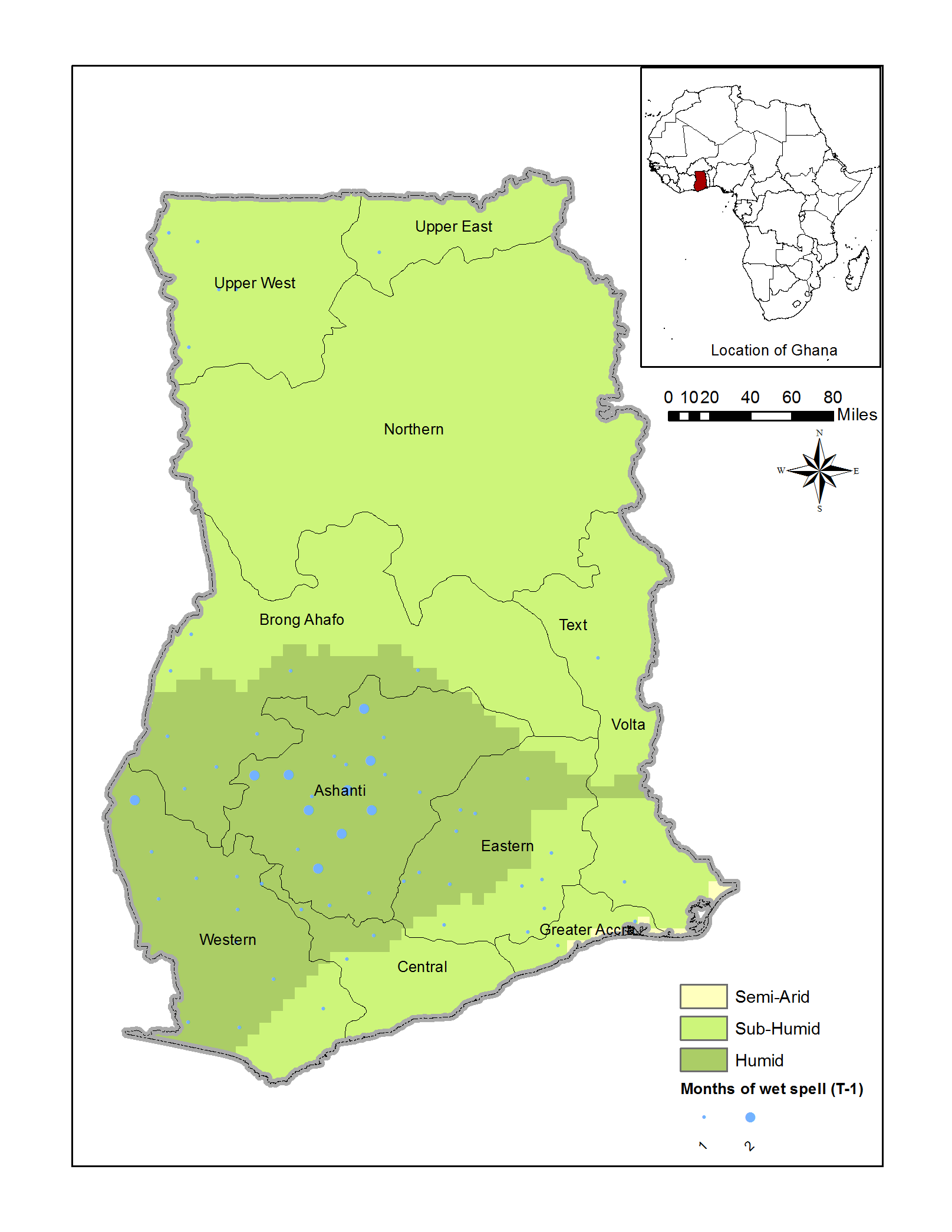

Supplement: S4 Fig — Summarized are the number of planting and growing season months at T-1 experiencing wet spell. The bigger the circle, the higher the number of months experiencing wet spell. (TIF) [file pone.0206415.s009.tif]

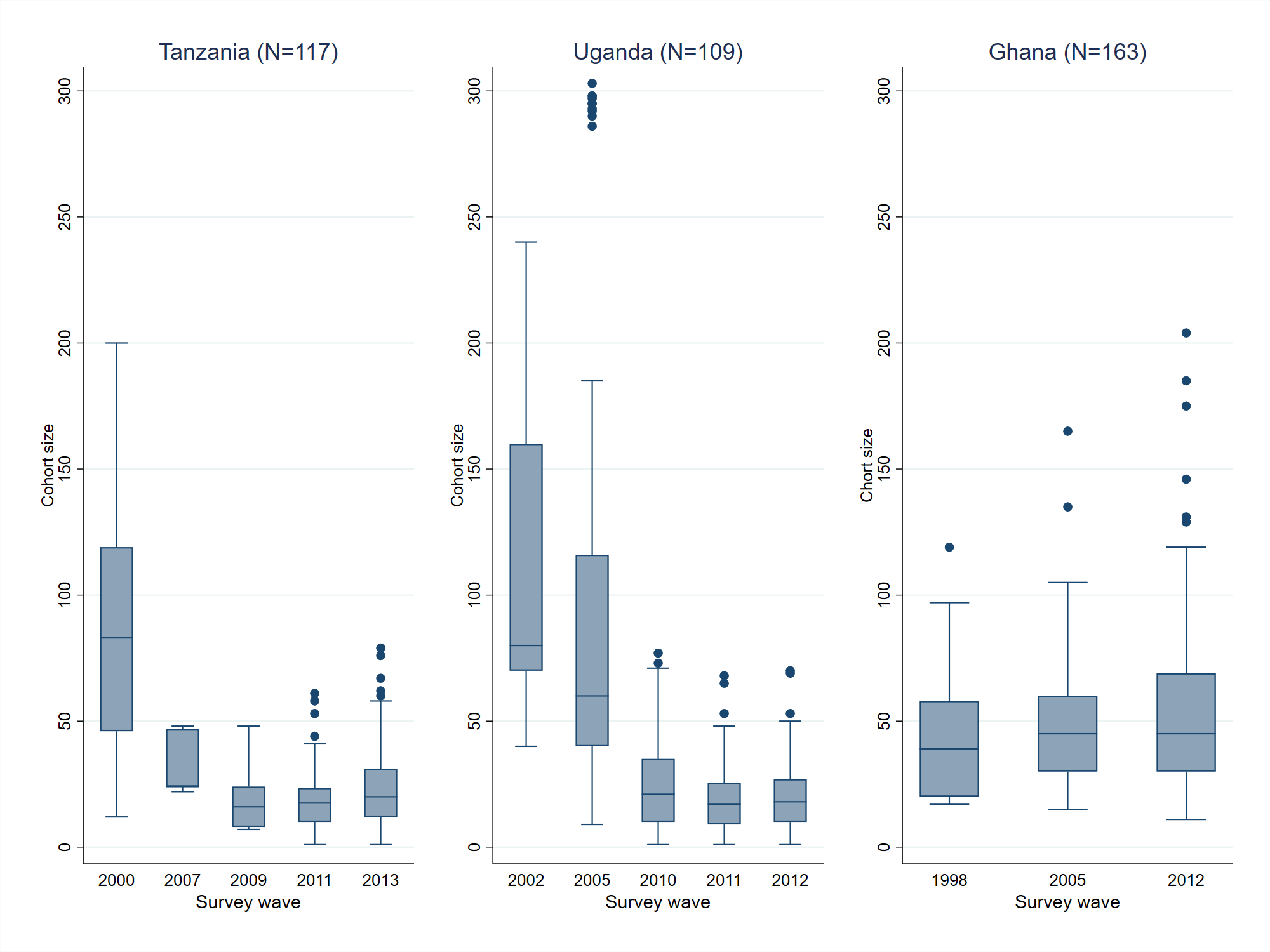

Supplement: S5 Fig — N = number of districts. (TIF) [file pone.0206415.s010.tif]

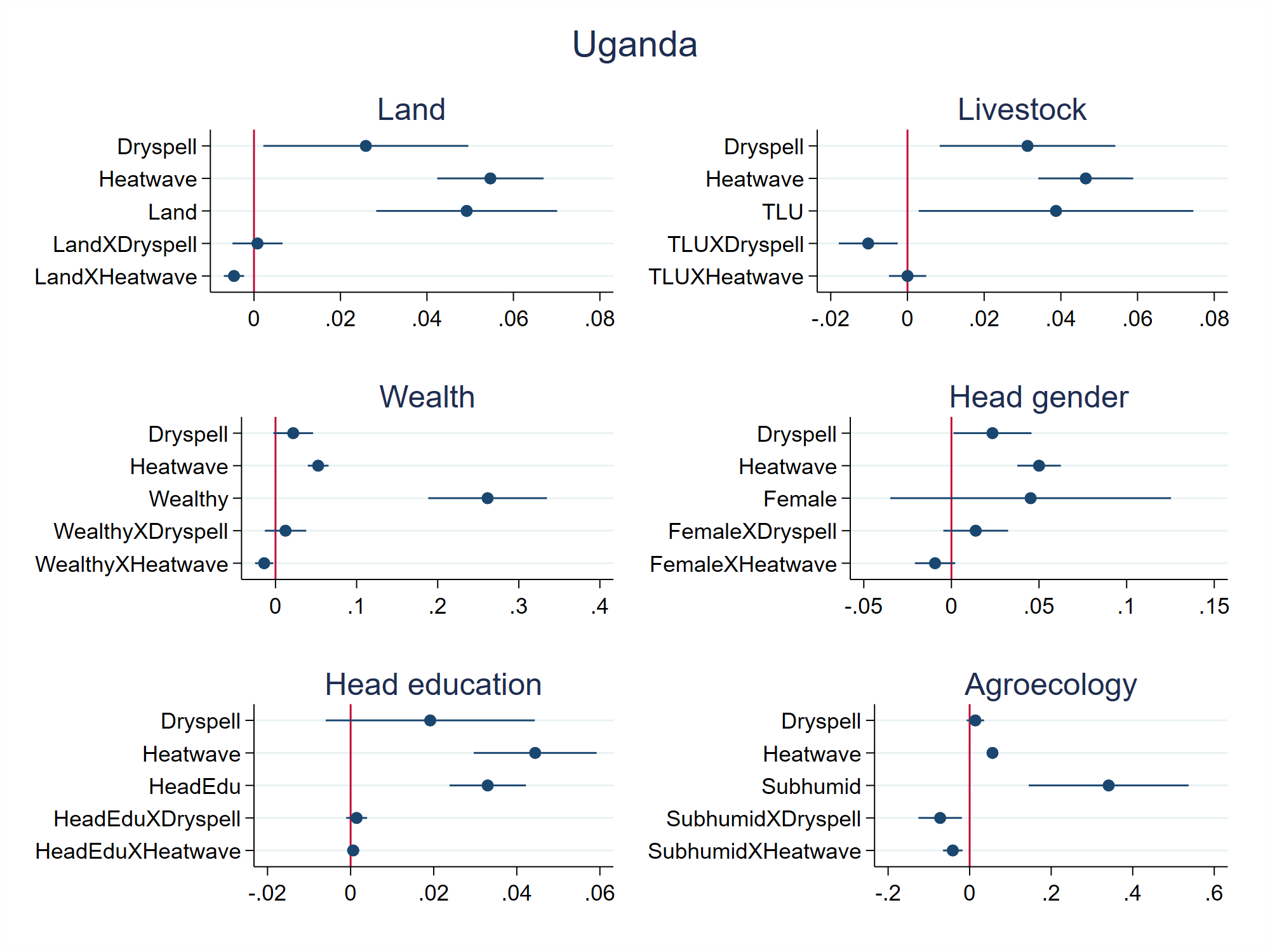

Supplement: S6 Fig — Plotted are weighted least squares coefficient estimates of along with 95% confidence bands from the fully parsimonious model. Only the coefficients of the variables of interest are shown. Land is measured in hectares; “Headedu” refers to education level of the household head; livestock asset is measured in “TLU”; “Wealthy” refers to households in the top tercile of durable-assets based wealth index. “X” represents an interaction term and a statistically significant coefficient of an interaction term implies differential effect. The omitted category is humid agro-ecology. (TIF) [file pone.0206415.s011.tif]

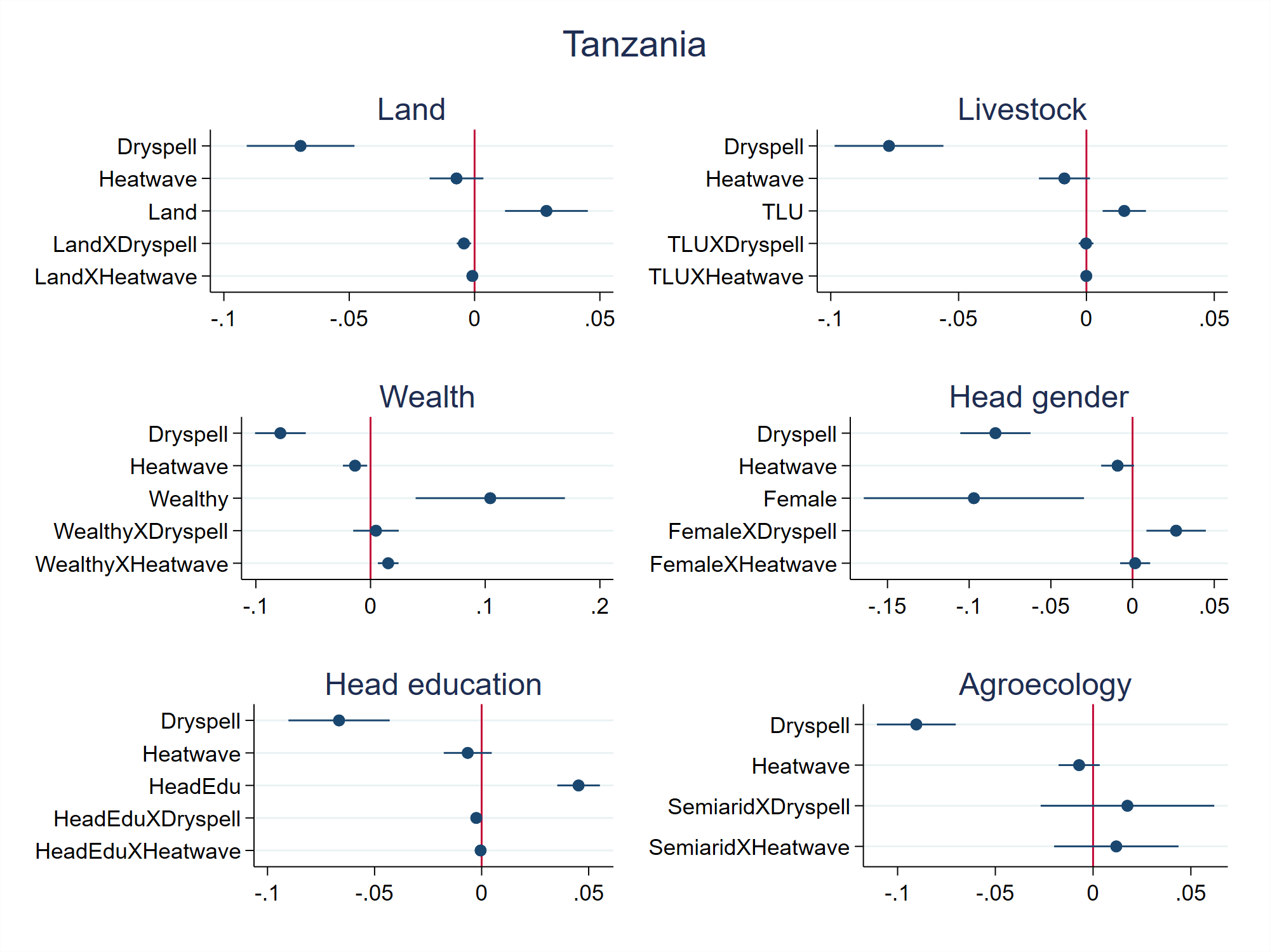

Supplement: S7 Fig — For notes, please refer to S6 Fig. The omitted category is sub-humid agro-ecology. (TIF) [file pone.0206415.s012.tif]

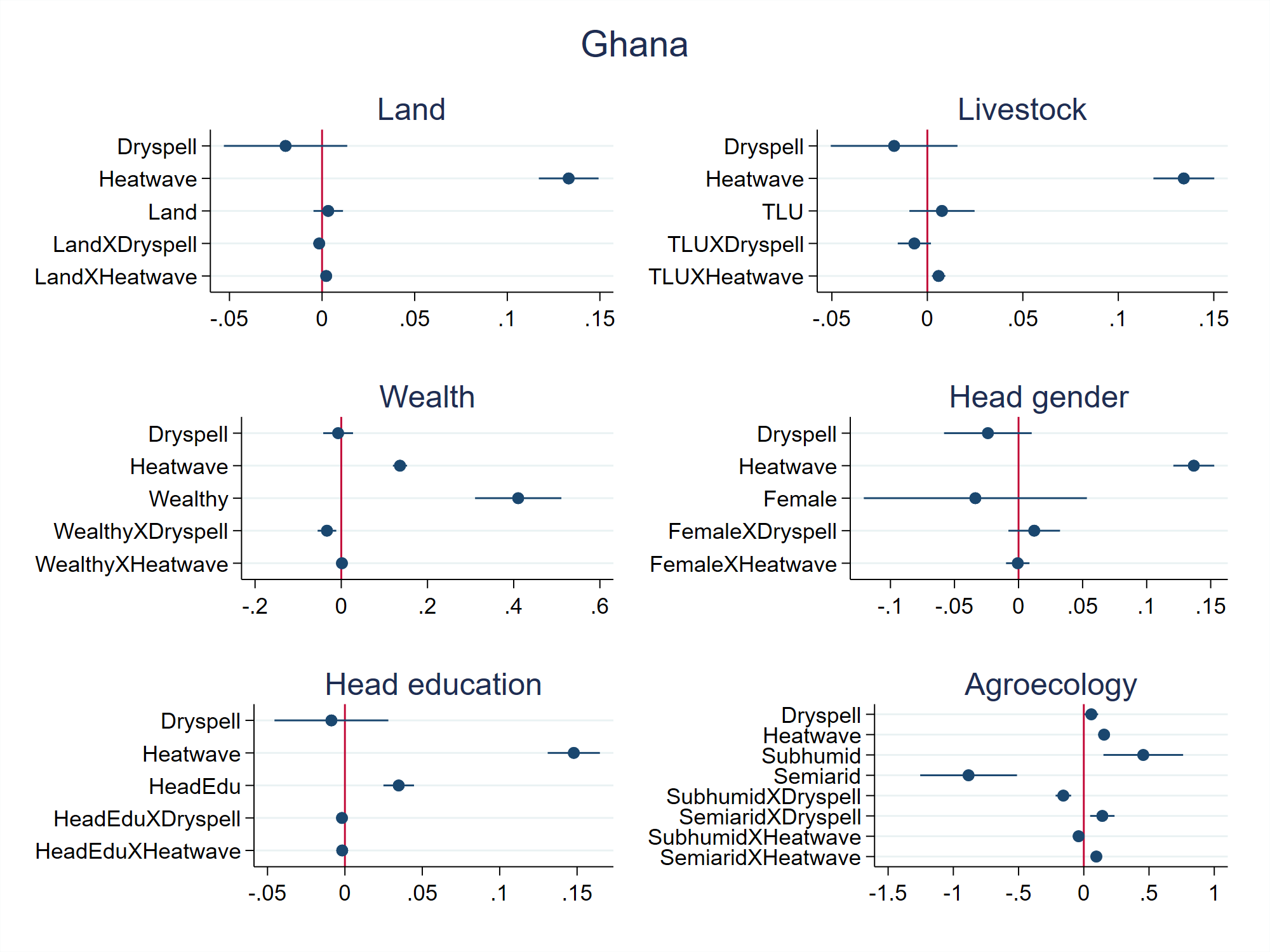

Supplement: S8 Fig — For notes, please refer to S6 Fig. The omitted category is humid agro-ecology. (TIF) [file pone.0206415.s013.tif]
